# Diet of the prehistoric population of Rapa Nui (Easter Island, Chile) shows environmental adaptation and resilience

**DOI:** 10.1002/ajpa.23273

**Published:** 2017-06-30

**Authors:** Catrine L. Jarman, Thomas Larsen, Terry Hunt, Carl Lipo, Reidar Solsvik, Natalie Wallsgrove, Cassie Ka'apu‐Lyons, Hilary G. Close, Brian N. Popp

**Affiliations:** ^1^ Department of Archaeology and Anthropology University of Bristol Bristol BS8 1UU Great Britain; ^2^ Leibniz‐Laboratory for Isotope Research, Christian‐Albrechts‐Universität Kiel 24118 Germany; ^3^ Clark Honors College and Department of Anthropology 1293 University of Oregon Eugene Oregon 97403‐1293; ^4^ Environmental Studies Program and Department of Anthropology Binghamton University Binghamton New York 13902; ^5^ Kon‐Tiki Museum Oslo 0286 Norway; ^6^ Department of Geology & Geophysics University of Hawai'i Honolulu Hawaii 96822; ^7^ Present address: Marine Biology, University of Hawai'I Kane‘ohe HI 96744; ^8^ Present address: Department of Ocean Sciences Rosenstiel School of Marine and Atmospheric Science, University of Miami, 4600 Rickenbacker Causeway Miami FL 33149

**Keywords:** amino acids, compound specific isotope analysis, ecology, radiocarbon, stable isotopes

## Abstract

**Objectives:**

The Rapa Nui “ecocide” narrative questions whether the prehistoric population caused an avoidable ecological disaster through rapid deforestation and over‐exploitation of natural resources. The objective of this study was to characterize prehistoric human diets to shed light on human adaptability and land use in an island environment with limited resources.

**Materials and methods:**

Materials for this study included human, faunal, and botanical remains from the archaeological sites Anakena and Ahu Tepeu on Rapa Nui, dating from c. 1400 AD to the historic period, and modern reference material. We used bulk carbon and nitrogen isotope analyses and amino acid compound specific isotope analyses (AA‐CSIA) of collagen isolated from prehistoric human and faunal bone, to assess the use of marine versus terrestrial resources and to investigate the underlying baseline values. Similar isotope analyses of archaeological and modern botanical and marine samples were used to characterize the local environment.

**Results:**

Results of carbon and nitrogen AA‐CSIA independently show that around half the protein in diets from the humans measured came from marine sources; markedly higher than previous estimates. We also observed higher δ^15^N values in human collagen than could be expected from the local environment.

**Discussion:**

Our results suggest highly elevated δ^15^N values could only have come from consumption of crops grown in substantially manipulated soils. These findings strongly suggest that the prehistoric population adapted and exhibited astute environmental awareness in a harsh environment with nutrient poor soils. Our results also have implications for evaluating marine reservoir corrections of radiocarbon dates.

## 1 INTRODUCTION

Rapa Nui (Easter Island, Chile) is frequently used as an exemplar of human social competition and an avoidable ecological disaster, in which rapid destruction of the native palm forest had devastating consequences for the island's environment and human population (e.g., Diamond, 2005). Recent archaeological research has brought such Malthusian claims into question: the arrival of the Pacific rat (*Rattus exulans*) shortly after the island's colonization may have extensively contributed to the palm forest's demise (Hunt, 2007) and with the use of fire the island was transformed into an agricultural landscape (Hunt & Lipo, 2006). Revised chronologies indicate settlement of Rapa Nui centuries later than previously supposed, with evidence for a more balanced use of the environment and a greater degree of human adaptability to a changing ecosystem than the “ecocide” model purports (Hunt & Lipo, 2006; Stevenson et al., 2015). Knowing past diets is crucial for understanding the impacts of human occupation on Rapa Nui. Despite the intrinsic connection between human behavior and the utilization of natural resources in this small and ecologically‐constrained island environment, prehistoric diets of the native islanders are still debated and poorly understood. Dietary evidence is spatially and temporally scattered, and its quality is affected by unfavorable preservation and taphonomic transformations; thus diet has been inferred from stable isotopic compositions.

Rapa Nui is a small (171 km^2^), remote volcanic island located in the south‐eastern Pacific initially colonized in the early 13^th^ century AD (Hunt & Lipo, 2006). Polynesian settlers introduced chicken (*Gallus gallus*) and the Pacific rat, but the island has no endemic mammals or land birds (Klemmer & Zizka, 1993). Introduced plants for subsistence purposes are mainly taro, sweet potato, and yam, but also *ti*, bananas, and sugarcane, and these are thought to have been cultivated in prehistoric times. Evidence for this comes from ethnohistorical accounts (e.g., Métraux, 1940; Roggeveen, 1908), surveys of the current flora (Flenley, 1993) and from microfossils in bioarchaeological and paleoecological studies (e.g., Dudgeon & Tromp, 2014; Horrocks et al., 2012a, 2012b; Horrocks & Wozniak, 2008). Archaeological evidence documents extensive use of lithic mulch in small‐scale rock gardens and planting enclosures (*manavai*) that served to increase soil nutrients, regulate soil conditions, and offset the effects of aridity and strong winds (Ayala‐Bradford, Lipo, & Hunt, 2005; Ladefoged et al., 2010). The importance of seafood in prehistoric Rapa Nui diet has been subject to debate. Evidence for marine resource use is typically elusive in the archaeological record on Rapa Nui due to the acidic volcanic soils, yet is demonstrated through abundant fish and marine mammal remains wherever deposits that preserve these kinds of materials are found (Hunt, 2007; Martinsson‐Wallin & Wallin, 1999). The presence of bone and stone fishhooks also attest to the creation of tools specifically for acquiring fish (Métraux, 1940). In addition, it has been suggested that microwear patterns on prehistoric human dental enamel are consistent with seafood consumption (Polet, 2011) and petroglyphs could suggest the cultural importance of fishing (Arana, 2014). However, estimates of the relative contribution of seafood to Rapa Nui diets vary considerably; for example while Métraux (1940) considers it to have been less important, Thomson (1891) described seafood as a principal dietary staple. Apparent variation in the archaeozoological record has been thought to relate to temporal differences and/or social stratification (e.g., Martinsson‐Wallin & Wallin, 1999). Existing studies of carbon and nitrogen stable isotope ratios in Rapa Nui prehistoric human collagen (Commendador, Dudgeon, Finney, Fuller, & Esh, 2013; Polet & Bocherens, 2015) suggest that seafood only contributed a minor part of dietary protein, and that the focus of subsistence was on consumption of chickens and rats. Similar patterns have been suggested elsewhere in prehistoric Pacific contexts (Richards, West, Rolett, & Dobney, 2009). Unlike on Rapa Nui, however, populations in those locations benefited from large introduced domesticates such as pigs and dogs, which could perhaps better satisfy nutritional demands than birds and small rodents. If marine resources were not utilized to a great extent on Rapa Nui, human subsistence could place substantial strain on other terrestrial resources.

Up to now, all stable isotope studies from Rapa Nui have used bulk collagen δ^13^C and δ^15^N values related to a reconstructed food web based on measured or hypothesized plant and faunal isotopic end members (Commendador et al., 2013; Fogel, Tuross, Johnson, & Miller, 1997; Polet & Bocherens, 2015). A disadvantage with this method is that it relies heavily on the appropriateness of selected reference values for the local environment. In their study of Rapa Nui bulk collagen isotopic composition, Commendador et al. (2013) used terrestrial end member δ^13^C (–20.5‰) and δ^15^N (6.5‰) values derived from published modern data for C_3_ plants from Pacific islands other than Rapa Nui, concluding that only a minor proportion of prehistoric diets came from marine resources. Although using modern bulk data is common practice in archaeology, the approach is problematic for Rapa Nui for several reasons. Firstly, significant environmental and land‐use changes have taken place on Rapa Nui over the past millennium (Flenley, 1993; Hunt & Lipo, 2011), so most plants grown under current conditions there are unlikely to be representative of prehistoric environments. Only 40% of the current flora on Rapa Nui is indigenous and an estimated 90% of Rapa Nui is now covered by grasslands, with post‐contact introduced grasses making up ∼80% of this Poaceae flora (Finot, Marticorena, Marticorena, Rojas, & Barrera, 2015). This is of particular relevance in isotopic studies as most invasive grasses on Rapa Nui are C_4_ plants in the subfamilies Chloridoideae and Panicoidae, which make up ∼60% of the grass species, whereas indigenous grasses were dominantly C_3_ plants (Finot et al., 2015). Secondly, Commendador et al. (2013) showed that the Rapa Nui humans exhibited particularly high δ^15^N values, an observation also noted by Fogel et al. (1997). Both studies suggested environmental factors, including the effect of seabird guano, as possible influences on this raised baseline. Szpak (2014) showed that δ^15^N values in particular are very sensitive to environmental variation, which demonstrates the importance of using reference values specific to a particular location if possible. Elsewhere archaeologically preserved cultivars have been used as proxies for evidence of past agricultural manipulation (Styring et al., 2015), but no such preserved macroremains suitable for isotope analysis have been found on Rapa Nui.

Here, we use unique carbon and nitrogen compound‐specific isotope analysis of amino acids (AA‐CSIA) in human and faunal remains to explore the extent of marine resource use in prehistoric Rapa Nui diets and to investigate the drivers of the elevated bulk δ^15^N values observed in previous studies. We also reevaluate prehistoric terrestrial end member δ^13^C values used to model bulk collagen isotopic compositions to account for possible variations in the local environment.

δ^13^C AA‐CSIA has been shown to be particularly helpful for distinguishing between marine and terrestrial diets (Corr, Sealy, Horton, & Evershed, 2005). Experiments have shown that dietary essential amino acids (EAAs) are routed to bone collagen with minimal carbon isotope fractionation, and must therefore reflect a consumer's diet (Howland et al., 2003; Jim, Jones, Ambrose, & Evershed, 2006). In ecological contexts, multivariate statistical analyses have been used to separate marine and terrestrial consumer groups using EAA δ^13^C “fingerprints” specific to dietary food sources (Larsen et al., 2013). Based on relative patterns between EAA δ^13^C (δ^13^C_EAA_) values, this fingerprinting approach provides an estimate for dietary protein resources that is independent from mass‐balance approaches, which must assume absolute δ^13^C values for terrestrial and marine end‐members.

δ^15^N values of individual amino acids allows for determination of trophic positions (TP) where δ^15^N values at the base of the food web are determined in analysis of consumers. In samples of consumer tissues, “source” amino acids (e.g., phenylalanine) retain the isotopic composition of nitrogen sources at the base of the food web, whereas “trophic” amino acids (e.g., glutamic acid) become ^15^N enriched with each trophic transfer (Chikaraishi et al., 2009; Hannides, Popp, Landry, & Graham, 2009; McClelland & Montoya, 2002; McClelland, Holl, & Montoya, 2003; Popp et al., 2007). The magnitude of ^15^N enrichment in trophic amino acids is most likely related to kinetic isotope effects associated with C‐N bond breakage, and the relative flux of the deamination products for each trophic amino acid (Chikaraishi, Kashiyama, Ogawa, Kitazato, & Ohkouchi, 2007). In contrast, the metabolic route for source amino acids, like phenylalanine, involves little formation or breakage of bonds to N atoms (e.g., Bender, 2007). Thus source amino acid δ^15^N values change little during trophic transfer (Chikaraishi et al., 2007, 2009). For these reasons, the trophic position of an organism can be determined using the difference in the δ^15^N values of glutamic acid (δ^15^N_*glu*_) and phenylalanine (δ^15^N_*phe*_). The major benefit of this technique is that it allows for direct paleodietary comparison in different temporal and geographical contexts, offering a refined estimate of TPs without requiring a full independent characterization of nitrogen isotopic composition at the base of the food web in the ancient local environment like that required for bulk collagen isotope analyses (Naito, Chikaraishi, Ohkouchi, Drucker, & Bocherens, 2013). This method is particularly relevant for Rapa Nui as it also allows for investigation of the δ^15^N baseline.

Our results indicate that marine protein formed a substantial component of the islanders' diet and do not support subsistence based on protein consumption predominantly from rats. The isotopic results further suggest that the crops consumed by the prehistoric population resulted from deliberate and sustained manipulation of infertile agricultural soils, indicating considerable adaptation to a barren island environment. These conclusions not only affect our understanding of resource use on the island, but also have implications for radiometric dating on human and faunal remains. Radiocarbon dating of such materials depends on reliable reconstructions of marine dietary input, to calibrate against marine reservoir effects (MRE) caused by incorporation of marine carbon with considerably older reservoir ages than terrestrial counterparts (Arneborg et al., 1999).

## 2 MATERIALS AND METHODS

### 2.1 Archaeological human, faunal, and botanical material

**Figure 1:**
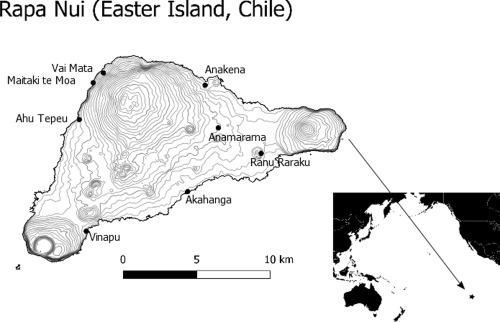
Map of Rapa Nui (Easter Island). Location of Rapa Nui with the archaeological sites Anakena (Ahu Nau Nau), Ahu Tepeu, and all other sampling sites shown. Grey contour lines show 20m elevation intervals

The majority of human (*n* = 10) and faunal (*n* = 25; rats, birds, fish, marine mammals) bone samples used in this study originate from 1986 to 1988 archaeological investigations at Anakena (Figure 1), carried out as a collaboration between the Kon Tiki Museum (Oslo) and the Museum in Hanga Roa (Rapa Nui), with a further two samples of human bone from excavations directed by Thor Heyerdahl in 1956 (see Skjølsvold, Wallin, & Martinsson‐Wallin, 1994). All human bone was sampled as rib fragments or phalanges from adult individuals, and with the exception of the 1956 material, no further information about age or sex was available. In addition to the human and faunal samples from Anakena, one sample of unburnt, preserved totora reed (*Schoenoplectus californicus*) associated with the burials in Grave 2 from Ahu Tepeu (Heyerdahl, Ferdon, Mulloy, Skjölsvold, & Smith, 1962) was analyzed along with a modern Totora sample collected as a part of Thor Heyerdahl's expedition to Rapa Nui in 1956. We also analyzed a preserved palm nut endocarp from Anakena. A summary is given in Table 1, and full list of materials analyzed is given in Supporting Information Table S1.

**Table 1:** Summary of sampled material grouped by type. For full details, see Supporting Information Table S1

Sample type	Origin	n	Site(s)
Terrestrial bird	Archaeological	1	Ahu Nau Nau
Bird	Archaeological	7	Ahu Nau Nau
Rat	Archaeological	16	Ahu Nau Nau, Anakena Dune
Fish	Archaeological	5	Ahu Nau Nau
Marine mammal	Archaeological	4	Ahu Nau Nau
Human	Archaeological	12	Ahu Nau Nau, Ranu Raraku, Ahu Tepeu
Totora reed	Archaeological	1	Ahu Tepeu
Totora reed	Historic	1	Unknown (Rapa Nui)
Soil	Archaeological	8	Anakena, Akahanga, Vai Mata, Ahu Nau Nau, Vinapu
Palm nut	Archaeological	1	Ahu Nau Nau
Soil	Modern	41	Maitaki Te Moa, Anakena, Anamarama
Plants	Modern	14	Denmark, Sri Lanka, Nigeria, Germany, Ecuador, Spain, Puerto Rica, Hawaii
Fish	Modern	3	Tropical North Pacific
Mussel	Modern	5	California

Permission for destructive analysis of the human remains was granted by the Kon Tiki Museum, in line with original permissions to export and analyze excavated material granted by Consejo de Monumentos Nacionales, Chile, for the excavations in the 1950s and 1980s. Further ethical approval for the study was granted by the University of Bristol Committee for Research Ethics.

Additional rat bones (*n* = 10) were obtained from excavations at Anakena Beach directed by Terry Hunt, Carl Lipo, and Sergio Rapu in 2004 to 2005 (Hunt & Lipo, 2006). These field studies focused on early deposits located in an area adjacent to Trench K, which formed part of the Kon‐Tiki Museum excavations in the 1980s. Excavated material from Anakena Trench K has previously been ^14^C dated to between AD1259 and AD1580 on the basis of two charcoal samples from different contexts (Skjølsvold et al., 1994). Dates from the nearby area excavated by Hunt and Lipo (2006) support this range of ages for the deposit. The sample from Ahu Tepeu (RN026) originates from a multiple grave in which one of the individuals has previously been ^14^C dated to 330 ± 150 BP (Heyerdahl et al., 1962).

### 2.2 Soils and modern reference materials

Forty one modern soil samples were chosen for bulk carbon and nitrogen isotopic analysis. Twenty nine soil samples were associated with *manavai* and twelve were associated with rock gardens. Samples were collected along north‐south transects across two *manavai* at Maitaki Te Moa and two at Anakena (for details see Hunt and Lipo, 2011). Thirteen samples were collected within the rock walls of *manavai* and the remainder outside these structures. Twelve soil samples from the Anamarama region were collected by Vitousek, Chadwick, Hotchkiss, Ladefoged, and Stevenson (2014) along an intensively sampled transect across two rock gardens (*n* = 8) separated by a tephra‐covered area with few surface rocks (*n* = 4). Details of sampling at Anamarama and the soil properties are given in Vitousek et al. (2014). Eight archaeological soil samples were analyzed; five from beneath fallen Moai statues and three from likely habitation contexts at Anakena and Vinapu.

We relied on both modern and archaeological training data for constructing the δ^13^C_EAA_ fingerprinting model. These training data comprised of 12 plant food samples (modern), three marine fish samples (modern), five marine mussels (modern), and six archaeological bone collagen samples from fish, marine mammals, and birds (Table 1 and Supporting Information Table S1).

### 2.3 Collagen extraction and bulk isotopic analysis

Collagen for stable isotope analysis was extracted from bone samples following a method similar to of Müldner and Richards (2005). Bone chunks (<1 g) were crushed and dissolved in 0.5M HCl until demineralized (approx. 3 days); when necessary, pH < 3 was maintained by addition of 0.5M HCl. Samples were rinsed with distilled water, 0.001M HCl was added and samples gelatinized at either 70°C for 48 h, or 90°C for 18 h. Finally, samples were filtered using glass wool packed into a Pasteur Pipette, and lyophilized.

The δ^13^C and δ^15^N values of lyophilized bulk collagen (0.5 mg) and all soil samples were determined using a Costech elemental combustion system (Model 4010) coupled to a ThermoFinnigan Delta Plus XP Isotope Ratio Mass Spectrometer (IRMS) through a Conflo IV interface, and are reported in standard δ‐notation relative to V‐PDB and atmospheric N_2,_ respectively and are reported in Supporting Information Table S2. Accuracy and precision were <0.2‰, as determined from multiple laboratory reference materials extensively calibrated using National Institute of Science and Technology reference materials and analyzed every 10 samples. Molar C:N ratios were determined to assess collagen preservation according to the commonly accepted range of 2.9–3.6 (DeNiro, 1985) and samples outside this range were excluded from further analysis.

### 2.4 Radiocarbon dating

Three samples of human bone from the current study (RN035, RN036, and RN037) were submitted for ^14^C AMS dating at the Center for Applied Isotope Studies at the University of Georgia. Collagen for ^14^C analysis was extracted from bone samples using 1N HCl, the residue filtered and rinsed with deionized water and collagen dissolved at 80°C for 6 hr. Lyophilized isolated collagen was graphitized in evacuated and sealed ampoules containing CuO at 575°C and the cleaned charcoal converted to CO_2_ by a similar procedure at 900°C in the presence of CuO. CO_2_ was cryogenically purified and then reduced to graphite (Vogel et al., 1984). Graphite ^14^C/^13^C ratios were measured using a CAIS 0.5 MeV accelerator mass spectrometer and normalized to measured values of Oxalic Acid I (NBS SRM 4990) as well as sample ^13^C/^12^C ratios. Measured radiocarbon dates were calibrated using OxCal 4.2 (Ramsey, 2009), with the SHCal13 atmospheric curve (Hogg et al., 2013) and Marine13 marine calibration curve (Reimer et al., 2013).

### 2.5 Preparation of samples for amino acid isotope analysis

A subset of 19 samples used for bulk collagen isotope analysis was chosen for compound‐specific amino acid (AA) δ^13^C and δ^15^N analysis (AA‐CSIA). This included all human samples with adequate collagen preservation, and a representative selection of possible food sources. In addition, the δ^15^N values of amino acids in the archaeological totora reed were also measured.

Samples for AA‐CSIA were hydrolyzed and derivatized according to the methods of Popp et al. (2007) and Hannides et al. (2009) for trifluoroacetyl/isopropyl ester derivatives and Larsen et al. (2015) for N‐acetylmethyl ester (NACME) derivatives. For preparation of trifluoroacetyl/isopropyl ester derivatives, 5–10 mg of collagen were hydrolyzed using trace‐metal grade 6M HCl and the hydrolysate purified using low protein‐binding filters and cation exchange chromatography. Purified samples were esterified using 4:1 isopropanol:acetyl chloride and derivatized using 3:1 methylene chloride:trifluoroacetyl anhydride. Finally, trifluoroacetyl/isopropyl ester derivatives were purified using solvent extraction (Ueda et al., 1989) and stored at −20°C for up to two weeks before analysis. Samples were prepared in batches of eight with an additional vial containing a mixture of 15 pure AAs purchased commercially (Sigma Scientific). For the NACME derivatives, 9–15 mg of dried material was hydrolyzed in 6M HCl (analytical grade HCl, Merck, Darmstadt, Germany). The samples were purified using cation exchange chromatography, dried and then methylated with acidified methanol and subsequently acetylated with a mixture of acetic anhydride, triethylamine, and acetone, forming N‐acetyl methyl ester derivatives. The esters were stored in ethyl acetate at −20°C until analysis.

### 2.6 Nitrogen isotope analysis of amino acids

The δ^15^N values of trifluoroacetyl/isopropyl ester derivatives of AAs were determined using gas chromatography combustion isotope ratio mass spectrometry (GC‐C‐IRMS, Hayes, Freeman, Hoham, & Popp, 1990). Specifically, we used an isotope ratio mass spectrometer (IRMS; Thermo Scientific Delta V or MAT 253) interfaced to a gas chromatograph (GC; Thermo Scientific Trace) fitted with a 60 m BPX5 *forte* column (0.32 mm internal diameter with 1.0 μm film thickness; SGE, Inc.) through a GC‐C III combustion furnace (980°C), reduction furnace (650°C), and liquid nitrogen cold trap. Helium (11.4 mL min^−1^) was used as the carrier gas. Immediately before analysis, samples were dried and redissolved in an appropriate volume of ethyl acetate. The analysis consisted of at least 6 injections for each sample, with norleucine and aminoadipic acid internal reference compounds co‐injected in each run. The suite of 15 pure amino acids was also analyzed every 3 injections to provide an additional measure of instrument accuracy. The isotopic composition of all pure amino acid reference compounds were previously measured using the bulk isotope technique described above. Nitrogen isotope values are reported in standard δ‐notation relative to atmospheric N_2_. For replicate injections of samples, amino acid δ^15^N standard deviations averaged 0.41‰ and ranged from 0.01‰ to 1.54‰ (Supporting Information Table S3). Uncertainty in calculations of trophic position, the fraction of marine protein in prehistoric human Rapa Nui diets and end member nitrogen isotopic composition was determined using propagation of errors and include all analytical errors and the uncertainty in the constants used (see Gelwicks & Hayes (1990), Blum, Popp, Drazen, Choy, & Johnson (2013) and Supporting Information Appendix A for details).

### 2.7 Carbon isotope analysis of amino acids

δ^13^C values of individual amino acids were also determined using GC‐C‐IRMS. The δ^13^C values of trifluoroacetyl/isopropyl ester derivatives were determined using an IRMS (MAT 253) interfaced with a Trace GC Ultra via a combustion furnace (1000°C) and ConFlo IV interface (Thermo Scientific). Samples were injected using a PTV (pressure/temperature/volume) injector, held at 40°C for three seconds, heated to 87°C (400°C min^−1^), heated again to 200°C and transferred at 200°C using a 1:10 split. Helium (1 mL min^−1^) was used as the carrier gas. Individual AAs were separated using a BPX5 *forte* capillary column (30 m × 0.32 mm internal diameter with 1.0 μm film thickness; SGE, Inc.). The oven temperature for the GC started at 40°C and was held for one minute before heating at 15°C min^−1^ to 120°C, then 3°C min^−1^ to 190°C, and finally 5°C min^−1^ to 300°C where it was held for an additional 10 min. Isotope values are reported in standard δ‐notation relative to V‐PDB and are reported in Supporting Information Table S4. Analysis consisted of at least 6 injections per sample with a perdeuterated *n*‐C_20_ alkane with a well characterized δ^13^C value co‐injected as an internal reference. The 15 AA reference suite was analyzed every 3 injections, and sample δ^13^C_AA_ values corrected relative to this AA suite following Silfer, Engel, Macko, and Jumeau (1991). The δ^13^C values of NACME derivatives of individual AAs were determined using a GC connected to a MAT 253 isotope ratio mass spectrometer via a GC‐III combustion interface (Thermo‐Finnigan Corporation) following the methods of Larsen et al. (2013) for analysis and correction for isotope effects during derivatization. A reference suite of 16 AA with known δ^13^C values was injected twice after every six injections of samples. Norleucine was used as an internal reference compound. Each sample was derivatized once and analyzed in triplicate.

For our samples the correction equation was: δ^13^C_AA_ = (δ^13^C_CSIA_ – (1 – X) × δ^13^C_ISO_)/X, where δ^13^C_AA_ is the corrected isotope value for the AA of interest, δ^13^C_CSIA_ is the isotope value initially determined by GC‐C‐IRMS, X is the mole fraction of amino acid C in each AA (vs. derivative C), and δ^13^C_ISO_ is the isotope value of the isopropanol added to each AA during esterification (the correction factor). Additionally, norleucine and aminoadipic acid reference compounds prepared with each sample batch were co‐injected with all samples and the reference suite of compounds analyzed, their δ^13^C values corrected using the above equation, and the results analyzed to establish instrument accuracy. For replicate collagen injections, average δ^13^C_AA_ standard deviations were 0.38‰ and ranged from 0.02‰–1.37‰ (Supporting Information Table S4). To account for inter‐laboratory differences, we applied the following calibration values to the Larsen data before statistical analyses: Leu; −0.86, Lys; −0.32, Phe; −3.75, Val; −0.76. The calibration is based on pairwise comparisons of three modern fish samples; Cg, Xg, and Lg (Arthur, Kelez, Larsen, Choy, & Popp, 2014).

Statistical analysis of δ^13^C_AA_ values was performed in R (version 3.2.1) or the OpenBUGS software package (Lunn, Thomas, Best, & Spiegelhalter, 2000). To create a fingerprinting model, we performed principal component analysis (PCA, R‐package vegan) on δ^13^C_EAA_ values centered to their sample means. Source diagnostic ability of δ^13^C_EAA_ fingerprints is based on patterns of isotopic difference among amino acids in a training set of reference samples, not absolute isotope ratios of the sample (Larsen, Taylor, Leigh, & O'Brien, 2009). The fingerprinting training dataset can therefore be drawn from a wide range of samples and settings. EAA are particularly well suited for reconstructing diets because metazoans cannot synthesize these essential nutrients *de novo* and are incorporated into bone collagen with little carbon isotope fractionation (Howland et al., 2003; Jim et al., 2006). We based our analysis on δ^13^C values of Leu, Lys, Phe and Val because they have previously been identified as the most informative EAA for distinguishing between marine and terrestrial food sources (Arthur et al., 2014; Larsen et al., 2013). We used samples that clustered nearby one another to define potential food groups. These food groups were used to calculate relative contributions of marine and terrestrial proteins to consumers with the software FRUITS version 2.0 (Fernandes, Millard, Brabec, Nadeau, & Grootes, 2014). For the groups with plant‐based proteins, we mostly included the most calorie dense plant foods; bananas, starchy vegetables and palm seed (Supporting Information Table S1). FRUITS is executed with BUGS, which is a software package for performing “Bayesian inference Using Gibbs Sampling” that includes an expert system for determining an appropriate Markov chain Monte Carlo scheme based on the Gibbs sampling.

## 3 RESULTS

### 3.1 Radiocarbon dates

The three dated bone samples, RN035, RN036, and RN037, yielded uncalibrated dates of 490 ± 25BP, 440 ± 25BP, and 110 ± 25BP respectively. Radiocarbon dates calibrated after correcting for marine reservoir effects (MRE) on the basis of estimated fractions of marine food in each individual's diet are shown in Table 2. A local reservoir correction (ΔD) value of −83 ± 34 was used, following Commendador, Dudgeon, Fuller, and Finney (2014) and the values available on the CHRONO database (http://calib.qub.ac.uk/marine). Results are given for marine dietary estimates based on linear interpolation of bulk δ^13^C collagen values, according to the terrestrial and marine end members used by Commendador et al. (2014), and on linear interpolation using end member phenylalanine δ^15^N values (δ^15^N_*phe*_) determined in the current study (see below). Samples RN035 and RN036 yielded calibrated radiocarbon dates consistent with the charcoal dates for Ahu Nau Nau (Anakena). The third sample (RN037), dates to a considerably later period, and is likely to represent a more recent disturbance.

**Table 2:** ^14^C data and calibrations. All calibrations were carried out using OxCal 4.2. For references and local reservoir correction (ΔD) values, see text

					Terrestrial calibration,(ShCal13)		Mixed marine/terrestrial calibration,(ShCal13 and Marine13)			
			% Marine protein estimates		95.4% confidence		δ^13^C marine estimate,95.4% confidence		δ^15^N*_*phe*_* marine estimate,95.4% confidence	
Sample ID	UG^a^ Lab ID	^14^C date BP	δ^13^C linear interpolation^b^	δ^15^N*_*phe*_* linear interpolation^c^	From (AD)	To (AD)	From (AD)	To (AD)	From (AD)	To (AD)
RN035	20801	490 ± 25	21.1%	38.4%	1417	1475	1445	1620	1480	1631
RN036	20802	440 ± 25	14.4%	56.4%	1443	1616	1458	1624	1532	1797
RN037	20803	110 ± 25	23.3%	49.4%	1697	1945	1815	1945	1880	1945
RN026^d^		330 ± 150	30.0%	54.6%	1424	1945	1470	1945	1507	1945

a UG = University of Georgia Center for Applied Isotope Studies.

b Terrestrial bulk δ^13^C end member value = −21‰, bulk marine δ^13^C end member value = −12‰, as defined by Commendador et al. (2014).

c Terrestrial δ^15^N_*phe*_ end member value = 22.7‰, marine δ^15^N_*phe*_ end member value =0.41‰.

d Heyerdahl et. al. (1962)

### 3.2 Bulk collagen, plant, and soil results

**Figure 2:**
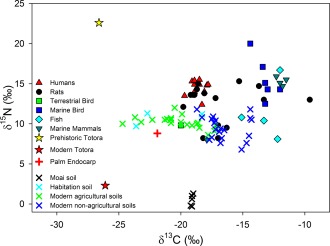
Plot of human, faunal, plant, and soil bulk δ^15^N and δ^13^C data from the current study. δ^13^C values of human collagen range from −19.7‰ to −17.8‰ (mean −18.6‰), and δ^15^N values range from 12.4‰ to 15.5‰ (mean 14.5‰). These values, along with the faunal data, are comparable with bulk collagen isotopic data from other Rapa Nui studies (Commendador et al., 2013; Polet & Bocherens, 2015)

Prehistoric human bulk collagen δ^13^C values range from −19.7‰ to −17.8‰ (mean −18.6‰) and δ^15^N values range from 12.4‰ to 15.5‰ (mean 14.5‰), and are shown graphically in Figure 2 and listed in Supporting Information Table S2. These values, along with similar faunal data, are comparable with bulk collagen results from other Rapa Nui studies (Commendador et al., 2013; Fogel et al., 1997; Polet & Bocherens, 2015). The δ^13^C and δ^15^N values of a prehistoric totora reed sample were −26.6‰ and 22.6‰, respectively, whereas the carbon and nitrogen isotopic values of a modern totora reed sample were −26.1‰ and 2.3‰, respectively. The δ^13^C value of a prehistoric palm nut endocarp was −21.9‰ and the δ^15^N value was 8.8‰. δ^13^C values of organic matter in the B horizon of archaeological soil samples from under Moai range from −19.2‰ to −19.0‰ (mean −19.1‰), and δ^15^N from −0.3‰ to 1.3‰ (mean 0.5‰). Soils excavated from Ahu Nau Nau and Vinapu, dating to approx. 1200–1400 AD, have δ^13^C values ranging from −23.6‰ to −18.3‰ (mean −21.5‰), and δ^15^N values from 9.7‰ to 11.3‰ (mean 10.7‰). These soils are thought to be A horizon soils from habitation contexts on the basis of soil structure, color, types of remains recovered, and contextual information (Skjølsvold et al. 1994, Kon‐Tiki Museum archives).

Modern soils collected within *manavai* and rock gardens have statistically lower δ^13^C values and higher δ^15^N values compared with soil samples from outside *manavai* walls and the rock gardens (mean δ^13^C in = −20.1 ± 2.1‰, δ^13^C out = −16.5 ± 1.4‰; *p* = <.001, Mann‐Whitney *U* test; mean δ^15^N in = 10.2 ± 0.7‰, δ^15^N out = 9.5 ± 1.5‰; *p* = .031, one‐tailed *t*‐test).

### 3.3 Results of δ^15^N AA‐CSIA

The nitrogen isotopic composition of amino acids was analyzed in 20 samples (Supporting Information Table S3). We targeted analysis of 13 amino acids, which we consistently measured in many samples. δ^15^N values of amino acids in collagen ranged from −32.0‰ to 28.7‰ (Supporting Information Table S3). Source amino acid (Gly, Ser, Phe, Lys) δ^15^N values of collagen ranged from −0.1 to 17.6‰ and trophic amino acids (Ala, Val, Leu, Pro, Asx, Glx) ranged from 8.8 to 28.7‰. Average source amino acid δ^15^N values of collagen were lower than trophic amino acids (9.0 vs. 15.7‰). The average source amino acid δ^15^N values of collagen were lowest in fish (4.9‰) relative to rats (8.5‰) and humans (11.3‰). On the other hand, the average trophic amino acid δ^15^N values of collagen were highest in fish (18.1‰) relative to rats (13.1‰) and humans (16.6‰). As noted by others (McCarthy et al., 2013; Nielsen, Popp, & Winder, 2015; Bradley et al., 2015) the δ^15^N values of Thr (–32.0 to 16.7‰) do not conform to either source or trophic amino acid groupings and appear to become depleted in ^15^N in higher trophic level organisms; as such we do not consider Thr in interpretation of our results. Source amino acid δ^15^N values of the archaeological totora reed ranged from 16.6 to 22.7‰ (average = 20.5‰) and trophic amino acids ranged from 19.5 to 29.0‰ (average = 23.9‰).

We used the δ^15^N values of the canonical source and trophic amino acids phenylalanine (δ^15^N_*phe*_) and glutamic acid (δ^15^N_*glu*_) to evaluate trophic positions. In addition, we focused on δ^15^N_*phe*_ values to model contributions of marine and terrestrial nitrogen sources to human and rat diets. For all collagen samples, average δ^15^N_*phe*_ values (8.7‰) were lower than average δ^15^N_*glu*_ values (18.0‰). Average fish δ^15^N_*phe*_ values (1.0‰) were lower than those for rats (10.4‰) and humans (11.5‰). Average human δ^15^N_*glu*_ values (18.1‰) were higher than those for fish (17.1‰) and rats (14.9‰). δ^15^N_*phe*_ values are linearly correlated with the average source amino acid (Gly, Ser, Lys) δ^15^N values (General Deming Regression (SigmaPlot 12.5), *r* ^2^ = 0.78) with a slope not significantly different than 1 (*p* = .0002). δ^15^N_*glu*_ values are also linearly correlated with the average trophic amino acid (Ala, Val, Leu, Pro, Asx) δ^15^N values (General Deming Regression (SigmaPlot 12.5), *r* ^2^ = 0.95) with a slope not significantly different than 1 (*p* = .0018). The δ^15^N_*phe*_ value of the totora reed was 22.7‰.

### 3.4 Results of δ^13^C AA‐CSIA

The carbon isotopic composition of amino acids was measured in 39 samples (Supporting Information Table S4). We targeted analysis of fourteen amino acids, which were consistently measured in most samples. δ^13^C values of amino acids in collagen ranged from −30.9 ‰ to 2.1‰ (Supporting Information Table S4). Collagen δ^13^C values of the nonessential amino acids (NEAAs) (Ala, Asx, Glx, Gly, Pro, Ser, Tyr) ranged from −24.3 to 2.1‰ and EAAs (Ile, Leu, Lys, Phe, Thr, Val) ranged from −22.9 to −5.8 ‰. Average δ^13^C_EAA_ values of collagen were lower than the average δ^13^C values of NEAAs (–22.4 vs. −15.8‰). The average δ^13^C_EAA_ values of collagen were higher in marine animals (–19.3 ‰) and humans (–22.2‰) than rats (–23.8‰) and terrestrial plants and birds (–24.2‰).

**Figure 3:**
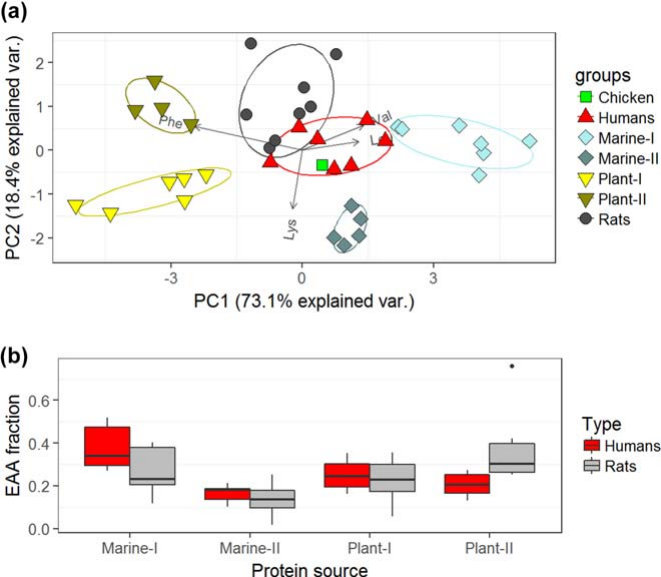
(a) Principal component analysis of normalized δ^13^C values of essential amino acids (lysine, phenylalanine, valine, leucine) from archaeological and modern samples. The statistical grouping illustrates that the Rapa Nui were omnivores relying on a mixture of marine and terrestrial protein sources. Marine‐I samples comprised of fish, marine mammal, and seabird, Marine‐II of mussels, Plant‐I of yam, common purslane, and leaves and tubers of sweet potatoes, Plant‐II of taro roots, bananas, bok choy and carob beans. (b) Proportional contributions of marine and terrestrial protein sources to humans (*n* = 7) and rats (*n* = 8) reconstructed from mean‐centered δ^13^C values of leucine, phenylalanine and valine of marine and terrestrial proteins. The boxes provide a 68% confidence interval and the whiskers provide a 95% confidence interval. See Supporting Information Tables S1 and S5 for identities of the protein sources and proportional protein contributions to each consumer

We applied δ^13^C_EAA_ fingerprinting to determine proportional contribution of terrestrial vs. marine EAA sources. We relied on both modern and archaeological training data for constructing the δ^13^C_EAA_ fingerprinting model. These training data comprised ten plant food samples (modern), three marine fish samples (modern), five marine mussels (modern), and six archaeological bone collagen samples from fish, marine mammals, and birds (Supporting Information Tables S1 and S4). Our PCA with mean‐centered δ^13^C values of Leu, Lys, Phe and Val revealed that the training data clustered in four distinct groups; two marine and two terrestrial groups. Marine‐I samples comprised of fish, marine mammal, and seabird, Marine‐II of mussels, Plant‐I of yam, common purslane, palm seed, and leaves and tubers of sweet potatoes, and Plant‐II of taro roots, bananas, and bok choy (Figure 3a). The principal component scores of most humans clustered closely to those of Marine‐I and the scores of most rats were intermediate between those of Marine‐I and Terrestrial‐I with the exception of a few rats being closer to Terrestrial‐II than Terrestrial‐I. The four food groups identified by the PCA were then used as end members in FRUITS to estimate proportional EAA contributions. In FRUITS, we used mean‐centered δ^13^C values of Leu, Phe and Val; we excluded Lys because according to the PCA it was the least informative EAA for distinguishing between marine and terrestrial protein sources. The mixing model data show that humans got more than half of their EAA from marine sources (54.3 ± 11.7%, *n* = 7, Figure 3b, Supporting Information Table S5) with Marine‐I making a greater contribution than Marine‐II (38.1 vs. 16.3%). The contributions from the two terrestrial groups were almost equal (25.0% for Plant‐I and 20.7% for Plant‐II). Marine EAA contribution for rats was 40.3 ± 11.8% (*n* = 8, Figure 3b, Supporting Information Table S5) with Marine‐I making a greater contribution than Marine‐II (26.6 vs. 13.6%). The contribution of Plant‐II was greater than that of Plant‐I (36.9 vs. 17.0%).

## 4 DISCUSSION

### 4.1 Diet inferred from bulk collagen isotopic compositions

Previous estimates of the fractional contribution of marine protein in prehistoric Rapa Nui human diets (ƒ_*marine*_) are largely based on bulk collagen δ^13^C values, and are founded in the values selected for mixing‐model end members and assumptions made about diet‐to‐collagen isotope fractionation (e.g., Commendador et al., 2013, 2014; Polet & Bocherens, 2015). In order to compare our compound‐specific techniques described below to this approach, we first estimated the proportion of marine protein in human diets using bulk δ^13^C data in a simple linear marine‐terrestrial two‐component mixing model (Phillips & Gregg, 2001), with a re‐evaluation of the most appropriate end member values. Average collagen δ^13^C values were calculated from all available isotopic results for humans (–18.4 ± 0.8‰, range −21.0 to −15.5‰, *n* = 144; Figure 2, Supporting Information Table S2, *n* = 11; Commendador et al. (2013), *n* = 41; Polet and Bocherens (2015) individuals 4 years and older, *n* = 92). For a marine end member we followed the recommendations of Ambrose, Butler, Hanson, Hunter‐Anderson, and Krueger (1997) and used the uncorrected average measured δ^13^C values of archaeological fish and marine mammal collagen from Rapa Nui (–12.4 ± 1.5‰, *n* = 52; Supporting Information Table S2. −13.1 ± 2.1‰, *n* = 9; Commendador et al. (2013) −12.2 ± 1.3‰, *n* = 43 data digitized from their Figure 4).

**Figure 4:**
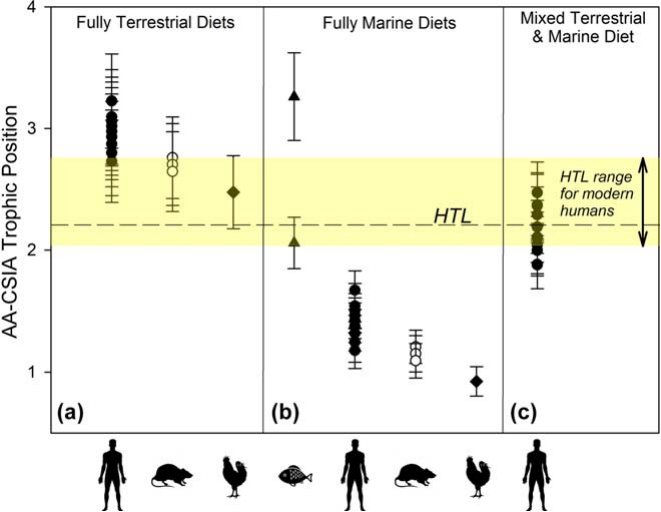
Trophic positions for humans, rats, marine fish and terrestrial bird. Trophic positions calculated using measured δ^15^N_*glu*_ and δ^15^N_*phe*_ values for human and faunal samples assuming fully terrestrial (a), and fully marine (b) diets. Trophic positions assuming a mixed diet (c) were calculated (Equation 3) using an estimate of ƒ_*marine (phe)*_ based on measured human δ^15^N_*phe*_ values as well as terrestrial and marine end member δ^15^N_*phe*_ values (Equation 7). Error bars denote standard deviations calculated using propagated errors (see Supporting Information Appendix A). Results are shown in relation to the range of trophic positions for modern humans, and the average global human trophic level (HTL; Bonhommeau et al., 2013)

Establishing a representative terrestrial end member δ^13^C value for Rapa Nui is more problematic. Richards et al. (2009) have used an assumed terrestrial end member δ^13^C value of −20 ± 1‰. This is based on global modern C3 plant data corrected for the ^13^C Suess effect and the assumption that collagen in a herbivore is enriched in ^13^C relative to plant material by a constant known amount (5‰) Fernandes, Nadeau, and Grootes, 2012. Although sugarcane, a C4 plant, was also likely present, there is evidence for only minor consumption of sugarcane on Rapa Nui in the prehistoric period (Dudgeon & Tromp, 2014), meaning that only C3 vascular plants need to be considered here.

Measured δ^13^C values of ancient humans on Rapa Nui place constraints on the terrestrial end member value; it must be lower than the most negative human δ^13^C value measured, otherwise the calculation results in *f_marine_* < 0 for some individuals, which violates mass balance. From all the available data, the lowest δ^13^C value for Rapa Nui human collagen was −21‰ (Polet & Bocherens, 2015), which, assuming a 5‰ diet‐to‐collagen ^13^C enrichment, suggests a maximum plant δ^13^C value of −26‰. Consequently, the higher terrestrial end member approximation used by others (e.g., Richards et al., 2009) may not be an accurate value for Rapa Nui. We suggest that a likely cause for this is that the local growing conditions for agricultural crops on Rapa Nui were unique and influenced plant δ^13^C values.

On the basis of sources cited above, we assume that major terrestrial plants in the prehistoric Rapa Nui human diet were taro, sweet potato, and yam grown in lithic mulch gardens and *manavai*. δ^13^C values of C3 vascular plants are sensitive to stomatal conductance and the δ^13^C value of atmospheric CO_2_ (Farquhar, Ehleringer, & Hubick, 1989). Increased soil moisture enhances leaf conductance that increases the partial pressure of CO_2_ within a leaf resulting in lower plant δ^13^C values (Farquhar et al., 1989). Agricultural manipulation of Rapa Nui archaeological soils increased their nutrient and moisture content (Ayala‐Bradford et al., 2005; Ladefoged et al., 2010; Vitousek et al., 2014). We therefore expect C3 plants grown in lithic mulch gardens and *manavai* with elevated soil moisture to have lower plant δ^13^C values than nonagricultural soils and plants on Rapa Nui. To test this assumption, we determined the isotopic composition of archaeological and modern soils and archaeological plant remains, and these differences apparently persist today. There are statistically lower δ^13^C values in modern *manavai* and rock garden soils compared with those of soils outside these agricultural features (Figure 2). However, modern soil samples on Rapa Nui are a poor representative of the Rapa Nui archaeological terrestrial end member δ^13^C value. Overall high δ^13^C values of our modern soil samples from Rapa Nui (mean −18.4‰, range −24.7 to −14.3‰, Supporting Information Tables S1 and S2) suggest at least some contribution from C4 plants regardless where they were collected, most likely due to a high proportion of modern invasive C4 grasses. Edwards and Still (2008) found that the distribution of C4 grasses on Hawaii correlated with areas of lower mean annual precipitation compared to their C3 counterparts. The lower proportion of C4 plants in modern soils within manavai and rock garden soils inferred from δ^13^C values therefore is consistent with higher soil moisture contents in agricultural soils that affect C3/C4 plant ratios (Edwards & Still, 2008; Ehleringer, Cerling, & Helliker, 1997). Historical land use changes, such as extensive sheep farming from ca. 1888 to 1953 (Flenley, 1993; Hunt & Lipo, 2011), has extensively altered the flora on Rapa Nui (Finot et al., 2015). Nevertheless, our data from modern soils suggest that plants grown in nonagricultural soils can yield different isotopic results from those grown in agricultural soils and that these differences are driven by soil moisture content.

No macroremains of archaeologically preserved cultivars suitable for isotopic analysis have been found on Rapa Nui, and the only preserved archaeobotanical samples available for this study were totora reeds and palm nut endocarps. The totora is a perennial sedge known to have been of cultural importance on Rapa Nui and in South America. Totora typically grow in muddy soils in freshwater marsh and riparian areas but is also found in cismontane and occasionally in desert environments (Munz, 1974). Although the growing conditions for the archaeological totora reed are unknown, it is observed today growing in moist soils around the fringe of freshwater crater lakes on Rapa Nui (e.g., Rano Raraku, Rano Kau, Rano Aroi, and Rano Aroi Iti; Horrocks et al., 2012a, 2012b, 2013, 2015). There is evidence for extensive cultivated rock gardens on the slopes adjacent to those lakes (e.g., Rano Kao, see Horrocks & Wozniak, 2008). Coexistence of totora and agricultural crops (e.g., taro, sweet potato, and yam) has been documented in sediment cores taken adjacent to Rano Raraku and Rano Kau (Horrocks et al., 2012a, 2012b). Since modern totora commonly grow in wet soils and there is paleobotanical evidence for the coexistence of totora and taro, sweet potato and yam, we assume that all of these plants grew under similar moist soil conditions. Therefore we suggest that the carbon isotopic composition of the archaeological totora as a representative terrestrial end member δ^13^C value. In fact, it is the only sample we analyzed that provides a terrestrial end member δ^13^C value (–26.6‰) that satisfies isotope mass balance and would yield *f_marine_* ≥ 0 for all ancient Rapa Nui. For comparison, the modern totora reed sample, collected by the Heyerdahl expedition in 1956, has a carbon isotopic composition (–25.6‰), similar to the archaeological sample when corrected for the δ^13^C value of atmospheric CO_2_ in 1956 based on measured ice core CO_2_ δ^13^C values from 1948 to 1962 (+0.5‰ correction, Francey et al., 1999). The archaeological totora reed carbon isotope ratio is also similar to δ^13^C values of modern taro and yam grown in agricultural plots in Oceania and Hawaii (taro = −25.2 ± 1.3‰, *n* = 17; yam = −26.8 ± 2.2‰, *n* = 9; Supporting Information Tables S1 and S2 (Allen & Craig, 2009; Chikaraishi & Naraoka, 2003; Kinaston et al., 2014)) when corrected for a Suess effect of +1.4‰ (Francey et al., 1999).

All archaeological soils and the prehistoric palm nut endocarp are enriched in ^13^C relative to the totora reeds analyzed, which is the relationship expected from nonagricultural soils or plants growing in well‐drained soil (palm) compared to plants grown in moist agricultural soil (totora, and presumably taro, sweet potato and yam). Importantly, using the mean δ^13^C values for archaeological soils corrected by 5‰ for the diet‐to‐collagen isotope fractionation in our calculations results in *f_marine_* < 0 for all ancient Rapa Nui humans, which violates mass balance. A similar calculation using the δ^13^C values for the palm nut endocarp as a terrestrial end member results in *f_marine_* < 0 for 95% of ancient Rapa Nui humans. We therefore use the archaeological totora reed δ^13^C value as our best estimate for our terrestrial end member, assuming a conservative uncertainty of ±2‰ and the commonly accepted value of 5‰ as the ^13^C enrichment of collagen relative to plant materials (Ambrose et al., 1997; but see also below).

Our two‐component linear bulk isotope mixing model is similar to previous approaches in the literature where only bulk isotope data has been available. Using our marine (–12.4 ± 1.5‰) and terrestrial (–26.6 ± 2.0‰; −21.6‰ when corrected for diet‐to‐collagen isotope fractionation) end members to calculate *f_marine_*, suggests that the prehistoric Rapa Nui humans obtained 35% (range 7–67%) of their food from marine sources with a propagated uncertainty of 5%. It should be noted that this estimate is not only dependent upon our chosen appropriate marine and terrestrial end member δ^13^C values, but is also dependent upon assumptions about the magnitude of diet‐collagen carbon isotope fractionation. The commonly used 5‰ diet‐to‐collagen fractionation value we assumed has been shown to vary by as much as 12.3‰ depending on diet type and quality (Ambrose et al., 1997; Howland et al., 2003). However, we have no evidence for variation in the diet‐to‐collagen fractionation value on Rapa Nui. Additionally, bulk collagen may be biased towards the protein part of the diet, which could also affect estimates of marine consumption. For example, in a study of prehistoric diets from the southern Mariana Islands, ƒ_*marine*_ estimates based on collagen δ^13^C values were considerably lower than those determined from carbon isotope analysis of bone apatite, thought to represent whole diet δ^13^C values, in two out of the three islands studied (Ambrose et al., 1997). A number of controlled feeding experiments have yielded similar results. Recently, Webb et al. (2017) showed that in pigs fed on diets with different amounts of marine protein, there was considerable variation in the relative difference between the whole diet and tissue δ^13^C values with even just a small change in marine input. Significantly, it was shown that this variation occurred on the individual amino acid level through differences in synthesis and routing of e.g., glycine. As a result, the authors concluded that bulk isotope values in e.g., collagen may significantly mask or even misrepresent the actual resources consumed in mixed marine/terrestrial diets.

In summary, even with the caveats outlined above, the results of bulk collagen δ^13^C values suggest that seafood comprised approximately 35% of ancient Rapa Nui human diet. This estimate is based on measured δ^13^C values of Rapa Nui ancient humans and seafood (fish and marine mammals) and assumes that the δ^13^C value of the archaeological totora reed is representative of the terrestrial plants grown in *manavai* and rock garden soils. Importantly, we suggest neither the δ^13^C value measured for Rapa Nui modern or archaeological soil samples, nor the archaeological palm nut endocarp, are appropriate terrestrial end members. All modern soils contain significant C4 plant remains as a result of the introduction of invasive C4 grasses and extensive historical changes in land use. The δ^13^C value of our archaeological totora is similar to that of agricultural crops grown throughout Oceania and Hawaii and which are known based on historical sources and paleobotanical research to be important foods for ancient Rapa Nui humans.

### 4.2 Dietary ƒ_*marine*_ estimate from essential amino acid δ^13^C fingerprinting

To refine dietary estimates, we applied AA‐CSIA to a subsample of human, faunal, and botanical remains. The δ^13^C_EAA_ fingerprinting results indicate that both marine and terrestrial protein sources made substantial contributions to the islanders' dietary protein (Figure 3a). To quantify the relative contributions of these resources, we employed a Bayesian stable isotope mixing‐model based on δ^13^C_EAA_ values (Fernandes et al., 2014). The mixing‐model results show approximately 54% marine protein (Marine‐I and Marine‐II combined) consumption by humans (Figure 3b, Supporting Information Table S5). The majority of the marine proteins derived from sources similar to fish or marine mammals (Marine‐I), while protein sources similar to shellfish make the smallest contribution (Marine‐II) (38.1 vs. 16.3% of total protein contributions). The contributions of the two terrestrial groups were almost equal. Notably, this approach considers only proteinaceous dietary sources, and relies only on the relative carbon isotopic patterns among EAAs. It is therefore independent from the bulk collagen δ^13^C mass balance described above and results in different estimates of marine resource usage. Both our δ^13^C_EAA_ and bulk collagen isotope results support the interpretation that marine resources were more important for the subsistence of prehistoric Rapa Nui people than previously assumed (Commendador et al., 2013; Polet & Bocherens, 2015). That said, the estimates of marine contributions were greater for δ^13^C_EAA_ than bulk isotope values. While δ^13^C_EAA_ dietary estimates are less biased than bulk isotope estimates owing to unknown bulk diet‐to‐collagen fractionation and isotope baseline values, the outcome of both approaches hinges on selecting the most representative prehistoric Rapa Nui food sources. We selected food sources based on ethnoarchaeological evidence, but dietary estimates could be refined further if we had information that could rank the importance of different plant or marine foods. We did not consider contributions from rats because their proteins comprise a mixture of both marine and terrestrial derived EAA sources.

### 4.3 Marine or terrestrial diet based on AA‐CSIA δ^15^N values

To further independently corroborate our dietary estimates based on bulk collagen δ^13^C and δ^13^C_EAA_ values, we used δ^15^N values of individual amino acids. Although several authors have suggested that the use of δ^15^N values of multiple trophic and source amino acids can provide greater confidence in trophic position determinations (Bradley et al., 2015; Décima, Landry, & Popp, 2013; Nielsen et al., 2015), here we focus on δ^15^N_*glu*_ and δ^15^N_*phe*_ values, which are more commonly used in archaeological studies (e.g., Naito, Honch, Chikaraishi, Ohkouchi, & Yoneda, 2010, 2013). It is often challenging to measure the δ^15^N values of a consistent suite of all trophic and source amino acids other than glutamic acid and phenylalanine in all collagen samples. However, our results show that the δ^15^N values of glutamic acid and phenylalanine are representative of the average δ^15^N values of trophic and source amino acids, respectively in those samples where a full suite of trophic and source amino acids could be analyzed. In addition, the difference between the δ^15^N values of trophic and source amino acids other than glutamic acid and phenylalanine is not available in vascular plants or when available the uncertainty in their δ^15^N values is much higher than for glutamic acid and phenylalanine (Chikaraishi, Ogawa, & Ohkouchi, 2010).

Differences in δ^15^N_*glu*_ and δ^15^N_*phe*_ values in consumers and primary producers must be taken into account for quantification of trophic position. Chikaraishi et al. (2009) showed that the trophic position of marine organisms (TP_*marine*_) could be quantified using Equation (1):

(1) $$TP_{marine}=\left(\frac{\delta^{15}N_{glu}-\delta^{15}N_{phe}+\beta_{marine}}{\Delta_{glu-phe}}\right)+1$$

where δ^15^N_*glu*_ and δ^15^N_*phe*_ are the isotopic composition of glutamic acid and phenylalanine in a consumer, respectively, and Δ_*glu‐phe*_ is the extent of ^15^N enrichment in glutamic acid relative to phenylalanine in the consumer with each trophic transfer, assumed here to be 7.6‰ for mammals (Naito et al., 2010), although recent work has shown that Δ_*glu‐phe*_ values of marine organisms may be up to 1.5‰ lower (Bradley et al., 2015; Nielsen et al., 2015). *β_marine_* defines the ^15^N enrichment in glutamic acid relative to phenylalanine in aquatic photoautotrophs, and is taken here as −3.4‰ (Bradley et al., 2015; Chikaraishi et al., 2009; Nielsen et al., 2015).

A similar quantification (Equation (2)) can be used to determine the trophic position of organisms feeding on C_3_ vascular plants (TP*_terrestrial_*, Chikaraishi, Ogawa, Doi, & Ohkouchi, 2011).

(2) $$TP_{terrestrial}=\left(\frac{\delta^{15}N_{glu}-\delta^{15}N_{phe}+\beta_{terestrial}}{\Delta_{glu-phe}}\right)+1$$

In this equation, the value for *β_terrestrial_* is 8.4‰ (Chikaraishi et al., 2011), where β_*terrestrial*_ defines the ^15^N content in glutamic acid relative to phenylalanine in terrestrial (i.e., vascular C3) plants. δ^15^N_*glu*_, δ^15^N_*phe*_ and Δ_*glu‐phe*_ are as defined above. For C_4_ plants, the β value is significantly lower than 8.4‰ (Chikaraishi et al., 2011). However, as noted historical and archaeological evidence indicates that the main crops grown on prehistoric Rapa Nui were C_3_ (Hunt & Lipo, 2006).

By definition, primary producers inhabit TP 1.0, whereas entirely herbivorous consumers have a TP of 2.0. Carnivores, consuming herbivores only, will display TPs of 3.0. In marine environments, TPs above 3.0 are common due to the more complex food webs observed among marine organisms. As omnivores, with mixed diets consisting of fish, meat, and terrestrial plant resources, humans would be expected to display TPs between 2 and 3, depending on the amount of fish and meat consumed. This is supported by an analysis of modern and recent historic human diets. Based on the Food and Agricultural Organization (FAO) national data on human food supply for 176 countries, Bonhommeau et al. (2013) demonstrated an average global human trophic level (HTL) of 2.21, with a range from 2.04 to 2.76. The highest values represent the pre‐1974 HTL of Iceland and reflect a high proportion of seafood and meat in the Icelandic diet. Earliest dietary data (∼1960) for populations in Oceania show HTL in close agreement with average global HTL (Fiji HTL = 2.15; French Polynesia HTL = 2.25; Kiribati HTL = 2.18; Samoa HTL = 2.14; Solomon Islands HTL = 2.10; Vanuatu HTL = 2.14). Although HTL in many island populations in Oceania showed increases up to 2.3–2.5 over the next 3–4 decades (see Supporting Information Table S2 in Bonhommeau et al., 2013), we speculate that these increases may be due to effects of globalization of the food supply and may not be characteristic of subsistence living in prehistoric contexts.

When using established wholly marine or terrestrial TP equations (Equations (1) and (2), respectively), our results show that the Rapa Nui prehistoric humans largely fall outside the HTL range of Bonhommeau et al. (2013), indicating mixed marine/terrestrial diets as more likely (Figure 4a,b). The archaeological fish (*n* = 2) displayed TPs of 2.1 ± 0.2 and 3.3 ± 0.4 using Equation (1) for marine organisms. For prehistoric rats, Equation (1) (fully marine diets) gave impossibly low TPs ranging from 1.1 ± 0.1 to 1.2 ± 0.1 (mean 1.2), whereas Equation (2) (fully terrestrial) gave TPs in the range of 2.7 ± 0.3 to 2.8 ± 0.3 (mean 2.7). The humans (*n* = 9) displayed TPs of 1.2 ± 0.2 to 1.7 ± 0.2 (mean 1.4) for fully marine diets, and TPs ranging from 2.7 ± 0.3 to 3.2 ± 0.4 (mean 3.0) assuming fully terrestrial diets. Although the marine fish plotted within their expected TP ranges, the results for rats and human samples were unrealistic both for fully marine and fully terrestrial diets (Figure 4a,b and Table 3), strongly suggesting mixed diets as more likely.

**Table 3:** Trophic positions calculated assuming terrestrial, marine, and mixed diets. TP= trophic position, SD = standard deviation. TP_*marine*_ (Equation 1), SD TP_*marine*_ (Supporting Information Equation S4), TP_*terrestrial*_ (Equation 2), SD TP_*terrestrial*_ (Supporting Information Equation S4), TP_*mixed*_ (Equation 3), SD TP_*mixed*_ (Supporting Information Equation S5). TP_*mixed*_ was calculated here using ƒ_*marine (phe)*_ estimates determined from Equation 7 (see also Supporting Information Appendix A).

Sample ID	Type	TP_*marine*_	SD TP_*marine*_	TP_*terrestrial*_	SD TP_*terrestrial*_	TP_*mixed*_	SD TP_*mixed*_
RN026	Human	1.18	0.15	2.73	0.34	1.88	0.20
RN033	Human	1.25	0.17	2.80	0.35	2.04	0.23
RN035	Human	1.42	0.15	2.98	0.36	2.37	0.25
RN036	Human	1.32	0.15	2.87	0.35	2.00	0.20
RN037	Human	1.51	0.14	3.06	0.37	2.29	0.23
RN038	Human	1.54	0.19	3.10	0.39	2.37	0.27
RN039	Human	1.67	0.16	3.23	0.39	2.47	0.25
RN040	Human	1.47	0.15	3.02	0.36	2.19	0.22
RN041	Human	1.38	0.14	2.93	0.35	2.11	0.21
	Average human	1.42	0.15	2.97	0.36	2.19	0.23
							
1307	Rat	1.21	0.14	2.76	0.34	1.68	0.16
1328	Rat	1.15	0.15	2.70	0.34	2.03	0.22
RN014	Rat	1.09	0.14	2.65	0.33	1.82	0.19
	Average rat	1.15	0.14	2.70	0.33	1.84	0.19
							
RN001	Terrestrial bird	0.92	0.12	2.48	0.30	‐	‐
							
RN007	Fish	3.26	0.36	‐	‐	‐	‐
RN023	Fish	2.06	0.21	‐	‐	‐	‐
	Average fish	2.66	0.29	‐	‐	‐	‐

### 4.4 Dietary protein estimated from AA‐CSIA δ^15^N values and mixed terrestrial/marine food sources

In order to estimate the fraction of marine protein (*f_marine_*) in human diets, we used an amino acid nitrogen isotope mass balance model that combines Equations (1) and (2) (see also Hebert et al., 2016; Styring, Fraser, Bogaard, & Evershed, 2014). This approach uses a *β_mixed_* value, which multiplies the estimated fraction of marine and terrestrial foods (*f_marine_* and 1‐ *f_marine_*, respectively) with their corresponding known marine or terrestrial β values.

(3) $$TP_{mixed}=\left(\frac{\delta^{15}N_{glu}-\delta^{15}N_{phe}+(1-f_{marine})(\beta_{terrestrial})+(f_{marine})(\beta_{marine})}{\Delta_{glu-phe}}\right)+1$$

where δ^15^N_*glu*_, δ^15^N_*phe*_, β_*marine*_, β_*terrestrial*_ and Δ_*glu‐phe*_ are as defined in Equations (1) and (2). Hence, calculation of TP_*mixed*_ requires knowledge of the proportions of marine and terrestrial protein in the prehistoric human Rapa Nui diets.

As a first approximation we assume that the HTL estimated from modern and historic human diets are representative of prehistoric humans on Rapa Nui and can therefore estimate the fraction of marine protein (*f_marine (HTL)_*) by rearranging Equation (3) to solve for ƒ_*marine*_:

(4) $$f_{marine-(HTL)}=\left(\frac{\Delta_{glu-phe}(TP_{HTL}-1)+\delta^{15}N_{phe}-\delta^{15}N_{glu}-\beta_{terrestrial}}{\beta_{marine}-\beta_{terrestrial}}\right)$$

The estimate of ƒ_*marine (HTL)*_ for prehistoric Rapa Nui derived from Equation (4) depends entirely on measured values of δ^15^N_*glu*_ and δ^15^N_*phe*_, known values of *β_marine_* and *β_terrestrial_*, and the assumption that the modern average global HTL is applicable to the prehistoric Rapa Nui population. We test this assumption below using an isotope mass balance of measured terrestrial and marine δ^15^N_*phe*_ values.

Our use of δ^15^N AA‐CSIA resulted in new estimates of marine input into human diets, substantially higher than previous estimates based on bulk collagen isotope analysis (Commendador et al., 2013; Polet & Bocherens, 2015) but in agreement with our independent estimates based both our bulk collagen δ^13^C and δ^13^C_EAA_ results. Using the average measured difference between δ^15^N_*glu*_ and δ^15^N_*phe*_ values in human bone collagen (6.6 ±1.1‰), and assuming that the average global HTL value of 2.21 is a reasonable estimate of trophic position for the prehistoric Rapa Nui population, indicates an ƒ_*marine (HTL)*_ of roughly 0.50 or ∼50% marine protein. On an individual basis, *f*_marine (HTL)_ values ranged from 0.44 ± 0.17 to 0.65 ± 0.16 (Table 4).

**Table 4:** Estimates of the fraction of marine protein (ƒ_*marine*_) in human diets. Equations used: ƒ_*marine (HTL)*_ (Equation 4), SD ƒ_*marine (HTL)*_ (Supporting Information Equation S6), ƒ_*marine (phe)*_ (Equation 7), SD ƒ_*marine (phe)*_ (Phillips & Gregg, 2001).

Sample ID	Type	ƒ_*marine (HTL)*_	SD ƒ_*marine (HTL)*_	ƒ_*marine (phe)*_	SD ƒ_*marine (phe)*_
RN026	Human	0.34	0.18	0.55	0.03
RN033	Human	0.38	0.18	0.49	0.04
RN035	Human	0.49	0.17	0.39	0.03
RN036	Human	0.43	0.17	0.56	0.03
RN037	Human	0.55	0.16	0.49	0.03
RN038	Human	0.57	0.18	0.47	0.04
RN039	Human	0.65	0.16	0.49	0.03
RN040	Human	0.52	0.16	0.53	0.03
RN041	Human	0.46	0.16	0.53	0.03
Average human		0.49	0.17	0.50	0.03

Our δ^15^N AA‐CSIA approach required an assumption of average prehistoric Rapa Nui HTL (Equation (4)); to confirm the validity of this approach, we determined if the implied terrestrial end member δ^15^N value for phenylalanine (δ^15^N_*terrestrial‐phe*_) was reasonable. Since the phenylalanine nitrogen isotopic composition of a consumer is a mix of nitrogen from its dietary sources (i.e., little or no isotope fractionation during uptake and metabolism in consumers), we can use a simple two‐component isotope mixing model:

(5) $$\delta^{15}N_{human-phe}=f_{marine}\delta^{15}N_{marine-phe}+(1-f_{marine})\delta^{15}N_{terrestrial-phe}$$

Rearranging Equation (5), we can solve for the δ^15^N_*terrestrial‐phe*_ value for prehistoric Rapa Nui that is consistent with an HTL of 2.21 and the known *β_marine_* and *β_terrestrial_* values.

(6) $$\delta^{15}N_{terrestrial-phe}=\frac{\delta^{15}N_{human-phe}-f_{marine-(HTL)}\delta^{15}N_{marine-phe}}{1-f_{marine-(HTL)}}$$

Here, we used our average δ^15^N_*phe*_ value determined for prehistoric Rapa Nui humans (δ^15^N_*human‐phe*_ = 11.6‰), the average measured marine fish value (δ^15^N_*marine‐phe*_ = 1.0‰), and the average ƒ_*marine (HTL)*_ (0.5) determined using Equation (4). This gave an estimated δ^15^N_*terrestrial‐phe*_ value of 22.2‰, which is high for a terrestrial environment but is remarkably close to the measured δ^15^N_*phe*_ value from the preserved archaeological totora reed from Ahu Tepeu (RN047, 22.7‰). A review of the currently available published δ^15^N values of phenylalanine from all marine and terrestrial plant sources (*n* = 97) reveal δ^15^N_*phe*_ values ranging from −6.9‰ to 18.7‰ (Bradley et al., 2015; Chikaraishi et al., 2010; Naito et al., 2010; Nielsen et al., 2015; Styring et al., 2014). For marine and terrestrial animals and humans (*n* = 665), the δ^15^N_*phe*_ values range from −7‰ to 20.1‰ (Bradley et al., 2015; Naito et al., 2010, 2013; Nielsen et al., 2015; Styring et al., 2015; Styring, Sealy, & Evershed, 2010). Only a small proportion (*n* = 14) of the latter are above the highest δ^15^N_*phe*_ value of humans among our dataset (RN035, δ^15^N_*phe*_ = 14.0‰), and no plant values are higher than the totora reed. However the terrestrial plant end member δ^15^N value implied by our totora δ^15^N_*phe*_ value is not unusually high. Szpak (2014) used experimental data and literature results of δ^15^N values for soil and plants distributed globally to demonstrate that soils and plants can be highly enriched in ^15^N when fertilized with seabird guano (δ^15^N values of plants and soils ranging from 25.5 to 44.7‰). Therefore it is not unusual or surprising to expect that agricultural crops grown in lithic mulch gardens and *manavai* that were potentially fertilized with seabird guano to have elevated δ^15^N values. The high δ^15^N_*terrestrial‐phe*_ value, which is consist with the δ^15^N_*terrestrial‐phe*_ value estimated using Equation (6) and the measured value of the archaeological totora reed, suggests that the use of a global average human HTL of 2.21 to calculate ƒ_*marine*_ is appropriate for Rapa Nui.

We propose that the archaeological Totora reed grew in an environment that closely represents plants cultivated in lithic mulch gardens and *manavai* on Rapa Nui. Our suggestion is consistent with the microfossil studies of Horrocks et al. (2012a, 2012b), which document wetland taxa coexisting with taro, sweet potato and yam. The biogeochemical reactions known to produce ^15^N enrichment in soils and plants are found in modern taro fields. For example, Penton et al., 2013, 2014) found coupled nitrification‐denitrification in soils vegetated with taro. In the flooded agroecosystems studies by Penton et al. (2013, 2014), rates of denitrification are very high (depth‐integrated porewater denitrification of up to 845 μmol N_2_ m^−2^ h^−1^) leading to nearly complete consumption of nitrate and little net isotope fractionation. However, in Hawaiian forest volcanic soils Houlton et al. (2006) measured soil nitrate δ^15^N values as high as 180‰ and that the extent of denitrification and ^15^N enrichment in soil organic matter was correlated with mean annual precipitation. They found greatest isotope discrimination associated with denitrification expressed in the plant‐soil cycle at levels of mean annual precipitation less than about 2500 mm year^−1^. Modern mean annual rainfall on Rapa Nui is less than about 2100 mm year^−1^ (Stevenson et al., 2015), thus if biogeochemical cycling in Hawaiian forest volcanic soils is a reasonable analog for soils in lithic mulch gardens and *manavai* then denitrification and ^15^N‐enrichment in plants growing in these soils is expected.

With an independent approximation of a δ^15^N_*terrestrial‐phe*_ end member based on plants grown in lithic mulch gardens and *manavai* soils, we rearrange Equation (6) to obtain an estimate of fraction of marine protein in human diets (ƒ_*marine (phe)*_) that does not depend upon an assumed HTL. Here we assume that the δ^15^N*_phe_* measured in the archaeological totora reed (22.7 ± 0.9‰) is a reasonable representative of ancient agricultural growth, and the marine end member δ^15^N_*phe*_ value used is the lowest measured marine fish in our study (0.4 ± 0.6‰). Uncertainty in values of ƒ_*marine (phe)*_ using this 2‐component isotope mixing model was determined by propagation of errors (Phillips & Gregg, 2001).

(7) $$f_{marine-\left(phe\right)}=\frac{\delta^{15}N_{human-phe}-\delta^{15}N_{terrestrial-phe}}{\delta^{15}N_{marine-phe}-\delta^{15}N_{terrestrial-phe}}$$

Using this approach, we obtained an ƒ_*marine (phe)*_ average of 0.50 and a range from 0.39 ± 0.03 to 0.56 ± 0.03. Returning to our initial TP calculations, we can use this average ƒ_*marine (phe)*_ value in Equation (3) to achieve an average TP_*mixed*_ for humans of 2.19 (Figure 4c). This again supports both the feasibility of calculating TPs from δ^15^N_*phe*_ end member values, and ƒ_*marine*_ for humans using the global average human HTL.

**Figure 5:**
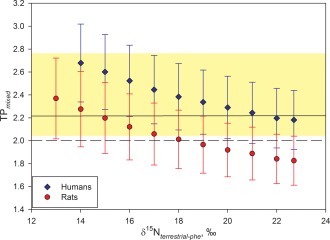
Plot of TP_*mixed*_ as a function of hypothetical δ^15^N_*terrestrial‐phe*_ values. We calculated ƒ_*marine (phe)*_ values based on average δ^15^N_*phe*_ values measured for fish, humans and rats and the hypothetical range of δ^15^N_*terrestrial‐phe*_ values shown using Equation 7. The calculated ƒ_*marine (phe)*_ values were used to determine the potential range of TP_*mixed*_ using the average measured Δδ^15^N_*glu‐phe*_ values for humans and rats and literature values for β_*marine*_, β_*terrestrial*_, and Δ_*glu‐phe*_ using Equation 3. The solid line is equal to the average global human trophic level (HTL), and the yellow rectangle shows the range of global human trophic level determined by Bonhommeau et al. (2013). The dashed line is equal to a TP_*mixed*_ value of 2.0

We believe that the archaeological Totora reed is a well‐preserved end‐member for this calculation and that its δ^15^N_*phe*_ value has not been diagenetically altered. Tremblay and Benner (2006) studied decomposition of plant materials under subaqueous marine conditions and found a small (2–5‰) increase in plant δ^15^N values. These authors found that amino acid composition and the %N as amino acids remained constant through the degradation experiment and therefore rejected the hypothesis that the small increase in δ^15^N values resulted from selective mineralization of ^15^N‐depleted, N‐rich biomolecules. In addition, for archaeological collagen, even samples with C:N outside the range typically expected for well‐preserved material can still consistently yield reliable δ^15^N_*glu*_ and δ^15^N_*phe*_ values (Naito et al., 2013, p. 2916). Nonetheless, we investigated the effect of different potential end member δ^15^N_*terrestrial‐phe*_ values on ƒ_*marine (phe)*_ and thus the calculation of TP_*mixed*_. Our choice of possible end members was based on the δ^15^N_*phe*_ values for humans as well as rats: we assumed that the highest hypothetical δ^15^N_*terrestrial‐phe*_ value is that measured for the archaeological totora reed (22.7 ± 0.9‰), and that the lowest hypothetical values are greater than or equal to the highest δ^15^N_*phe*_ value measured in humans (14‰) and rats (13‰). Lower values would result in calculations of f_*marine*_ < 0, which violates mass balance. Using Equation (7) and the potential range of δ^15^N_*terrestrial‐phe*_ values and the average δ^15^N_*phe*_ values measured for fish (1.0 ± 0.9‰), humans (11.6 ± 1.2‰), and rats (11.4 ± 2.9‰), ƒ_*marine (phe)*_ values calculated ranged from 0.19 ± 0.05 to 0.51 ± 0.03 for humans, and 0.22 ± 0.15 to 0.57 ± 0.08 for rats. We then used these ƒ_*marine (phe)*_ values to determine TP_*mixed*_ from Equation (3), using the average Δδ^15^N_*glu‐phe*_ values (and ranges) for humans and rats, and literature values for β_*marine*_, β_*terrestrial*_ and Δ_*glu‐phe*_. The effect of δ^15^N_*terrestrial‐phe*_ values on calculated TP_*mixed*_ is summarized in Figure 5. This calculation indicates that using even the widest possible range of δ^15^N_*terrestrial‐phe*_ values still suggests at least 20% marine protein in the ancient Rapa Nui human diet.

Although the higher marine estimates shown here challenge the conclusions of previous bulk collagen studies, our results are not inconsistent with archaeological evidence for fishing and marine consumption on the island. Excavations of Anakena by Hunt and Lipo (2006) and Hunt (2007) show a significant fraction (28–29%) of all preserved faunal remains to be from fish. Similarly, in a recent summary Arana (2014) details ethnohistorical sources that describe a range of boats, canoes, and totora reed floats used by the Rapa Nui population for fishing purposes. These, and extensive representation of fish and marine species, can also be seen on petroglyphs and as stone statues (e.g., Heyerdahl, 1958; Heyerdahl et al., 1962; Lee, 1993). It has been argued by some (e.g., Polet & Bocherens, 2015) that Anakena may have represented a more specialized or higher status site than other locations on the island resulting in systematically higher nitrogen and carbon isotope values. The notion that status is related to food consumption is difficult to justify given that Anakena is also situated in a location with easy access to marine resources. Given the lack of resource access restriction and the very localized structure of prehistoric Rapa Nui communities (Dudgeon, 2008; Lipo, Hunt, & Hundtoft, 2010; Morrison, 2012), the differences in fish consumption likely reflect relative proximity to these resources and not status.

### 4.5 Implications of results for human consumption of rats

Our study does not support the hypothesis proposed elsewhere that rats formed a major dietary source (Commendador et al., 2013). An independent estimate of fraction of marine protein in prehistoric Rapa Nui human and rat diets (ƒ_*marine (phe)*_; i.e., not assuming TP of the consumer) can be made using end member δ^15^N_*phe*_ values of the archaeological totora reed (22.7 ± 0.9‰) and the lowest measured marine fish (0.4 ± 0.6‰) using Equation (7). These values of ƒ_*marine (phe)*_ can then be substituted for ƒ_*marine*_ in Equation (3) to evaluate the relative difference in calculated TP_*mixed*_ for humans and rats (Table 3), and thus allow us to discern the probability of a direct dietary connection between rats and humans.

The modeled TP_*mixed*_ values in Figure 5 allow us to evaluate the suggestion that rats were a major dietary source of protein for humans (e.g., Commendador et al., 2013). Gut contents of the Pacific rat (*R. exulans*) studied on Hawaii revealed a diet of roughly 60% plant matter and 40% arthropod (Shiels et al., 2013); considering any proportion of omnivory, solutions resulting in rat TP_*mixed*_ values less than 2.0 must be eliminated. In addition, palm endocarps from the extinct relative of the *Jubaea* palm found in archaeological contexts on Rapa Nui all show distinctive rat gnaw marks (Hunt, 2007) indicating that prehistoric Rapa Nui rats ate at least some noncultivated plants most likely with lower δ^15^N_*terrestrial‐phe*_ values, which also are consistent with rat TP > 2.0 (Figure 5). Therefore we estimate that the most probable range of rat TP_*mixed*_ values is 2.01 ± 0.26 to 2.37 ± 0.35. Still considering the wide plausible range of end member δ^15^N_*terrestrial‐phe*_ values for both humans and rats, the maximum difference in TP_*mixed*_ values between humans and rats is <0.37. This suggests that that rats were feeding at a similar albeit slightly lower TP to humans making them incompatible as a major dietary source, since we would otherwise expect a TP difference greater than that observed. Thus, even if rat diet included a higher proportion of (noncultivated) terrestrial plants, their calculated TP_*mixed*_ converge on that of humans. Although it is likely that the Rapa Nui humans consumed rats to some degree, our data show they could not have been a major protein source for the individuals sampled here. The previous studies that suggested high rat consumption based this conclusion on a lack of other suitable food sources with sufficiently high δ^15^N and low δ^13^C values to explain the human data (Commendador et al., 2013). Our bulk data are very similar to that presented in other Rapa Nui studies (Commendador et al., 2013; Polet & Bocherens, 2015); however, with the addition of our TP calculations and re‐evaluation of appropriate bulk terrestrial end member values, we propose that neither the data presented here nor elsewhere support a high degree of rat consumption among humans.

### 4.6 Implications of results for radiocarbon dating

Higher marine dietary estimates obtained from the AA‐CSIA approaches affect calibrations of ^14^C dates from archaeological samples. When dating samples of human or faunal collagen from organisms that have had marine dietary input, the extent of the marine reservoir effect (MRE) must be assessed. This is because such samples will incorporate marine carbon with considerably older reservoir ages than terrestrial counterparts (Arneborg et al., 1999). For example in this study, when *f_marine_* is estimated based on δ^15^N_*phe*_ values rather than on linear interpolation of bulk δ^13^C collagen values according to the terrestrial and marine end members used by Commendador et al. (2014), the latest possible calibrated ^14^C date for one individual (RN036) was made more recent by 173 years (Table 2). For relatively short chronologies of human settlement, use of AA‐CSIA to estimate MRE may be particularly important, as collagen can be argued to provide more direct evidence of human occupation than botanical samples, particularly for secondary inhumations or, as on Rapa Nui, where human remains are often found in cave sites that may have limited stratigraphic information.

### 4.7 Soil management as a source of elevated δ^15^N values and its implications

Human collagen from Rapa Nui displays high bulk δ^15^N values that are particularly confounding to dietary reconstructions (Figure 2). Our TP calculations confirm that high δ^15^N values observed in bulk human collagen do not derive from high TPs, but rather from the base of the food web. The δ^15^N_*phe*_ values of marine fish average 1.0 ± 0.9‰, hence, the elevated δ^15^N_*phe*_ values observed in human collagen (Supporting Information Table S3) must come from terrestrial sources with δ^15^N_*phe*_ values considerably higher than those found in humans. The measured δ^15^N_*terrestrial‐phe*_ value of 22.7‰ for the archeological totora sample is high but is consistent with our calculations of ƒ_*marine*_ based on multiple independent approaches. This therefore constrains a maximum prehistoric end member δ^15^N_*phe*_ terrestrial value for Rapa Nui. It is notable that a modern totora sample from the island, collected in 1956, has a considerably lower bulk δ^15^N value of 2.3‰ than the archaeological sample (δ^15^N = 22.6 ‰), which may serve to show the substantial change in the island's environmental conditions since prehistoric times.

In order to investigate the prehistoric environment further, we measured values of δ^15^N and δ^13^C in nonagricultural archaeological soils from across the island as a proxy for past environmental conditions (Supporting Information Table S2). The soil sample δ^15^N values showed sizeable variability both spatially and contextually across the island (0.3–11.3‰). Additionally, an archaeological palm nut endocarp from Anakena had a δ^15^N value of 8.8‰. Although the soil from habitation contexts and the palm nut endocarp are enriched in ^15^N relative to the soils underlying Moai, their δ^15^N values are still lower than those of human collagen and cannot be representative of the environments in which agricultural crops grew.

The low δ^15^N values of the nonagricultural archaeological soil samples show that the elevated values observed in human material are not simply a result of general Rapa Nui environmental conditions. As described above, a terrestrial end‐member δ^15^N_*terrestrial‐phe*_ value of 22.7‰, based on the archaeological totora reed, is consistent with estimates of high (∼50%) marine components of prehistoric Rapa Nui diets, as converged upon by our several independent calculations. Since the totora reed and the palm nut endocarp were the only archaeological plant materials recovered, we separately determined a conservative estimate for a likely range of δ^15^N_*terrestrial‐phe*_ values, using instead our *lowest* calculation of ƒ*_marine_* (0.35 ± 0.05), which derives from our bulk isotope mixing model and measured bulk collagen δ^13^C values. Using this estimate as a lower limit for ƒ_*marine*_ results in terrestrial end member δ^15^N*_terrestrial‐phe_* values greater than about 18‰ (18–22.2‰) and corresponds to human TP_*mixed*_ values from 2.18 ± 0.26 to 2.38 ± 0.29 (Figure 5). These conservative δ^15^N_*terrestrial‐phe*_ values are higher than any of our measured archaeological soil or palm nut endocarp δ^15^N values, supporting our hypothesis that prehistoric Rapa Nui humans manipulated agricultural garden soils, resulting in higher nitrogen isotope ratios of cultivars that were inherited by human consumers. We therefore speculate that prehistoric islanders created conditions in the soil of their agricultural plots that had nitrogen biogeochemical conditions similar to that in which the archaeological totora reed grew.

We propose that the elevated terrestrial δ^15^N values are a result of substantially different soil N cycling processes in anthropogenically altered archaeological soils in the lithic mulch gardens and *manavai* that favored denitrification or ammonia volatilization (Szpak, 2014). Soil moisture levels are higher in modern lithic mulch gardens and *manavai* compared to natural soils (Hunt & Lipo, 2011), which would favor denitrification (e.g., Houlton et al., 2006). Furthermore, numerous studies have demonstrated that soil nutrients are higher in Rapa Nui lithic mulch gardens and *manavai* compared to natural soils, in part as a result of human interventions (Hunt & Lipo, 2011; Ladefoged et al., 2010; Louwagie, Stevenson, & Langohr, 2006; Vitousek et al., 2014). In addition, modern *manavai* and rock garden soils have significantly higher δ^15^N values compared to adjacent nonagricultural soils despite the predominance of introduced plants. Manuring can markedly increase the ^15^N content of agricultural soils, and this ^15^N enrichment is passed on through the food chain to humans (Fraser et al., 2011). The use of seabird guano for manure has been proposed as a mechanism for the elevated δ^15^N values observed in Rapa Nui human collagen (Commendador et al., 2013; Fogel et al., 1997), and it has been demonstrated that guano as a fertilizer can have a substantial effect on δ^15^N values in plants (Szpak, 2014; Szpak, Longstaffe, Millaire, & White, 2012). The *manavai* were likely used as deposits for household waste (Hunt & Lipo, 2011), and chicken husbandry may also have contributed to the elevated δ^15^N values observed.

## 5 CONCLUSIONS

Our results of carbon and nitrogen isotopic analysis of individual amino acids show that in our samples, seafood made up about half of the protein in human diets, which is considerably higher than previous estimates based on bulk data with similar isotopic compositions. Our estimates are consistent across four independent modelling approaches. Additionally, we show that rats are unlikely to have made up a significant source of human dietary protein. These results may demonstrate a more balanced subsistence strategy, which is less likely to have placed unnecessary strain on natural terrestrial resources. Furthermore, the more accurate estimation of marine input in human and faunal diets allows for improved MRE corrections of ^14^C dates. On Rapa Nui, the dating of the initial colonization of the island has been subject to extensive debate (Hunt & Lipo, 2006), and a detailed chronology is essential to understanding the impact of human settlement on the environment. The ^14^C dates from human and faunal remains have typically been considered less reliable due to MREs (Lipo & Hunt, 2016) despite their potential as more direct evidence for human occupation.

Significantly, our nitrogen isotopic results also suggest cultivation of agricultural crops in lithic mulch gardens and *manavai*, as documented in the archaeological record, was the source of the high δ^15^N values observed in prehistoric human remains. This is further supported by our analysis of ancient and modern soils from both agricultural and noncultivated contexts. We do not know if biogeochemical conditions in these agricultural plots favored denitrification or ammonia volatilization, or if manuring through bird waste produced the high cultivated plant δ^15^N values that were inherited by prehistoric humans. Regardless, these gardens required considerable effort in transporting the stones required to construct and maintain *manavai* and mulched areas (Ayala‐Bradford et al., 2005; Bork, Mieth, & Tschochner, 2004), attesting to the effort invested in cultivating terrestrial resources. Burning of the native forest would have temporarily increased soil fertility on Rapa Nui, but over time the soils would have lost fertility (Hunt & Lipo, 2011). Our results point to concerted efforts to manipulate agricultural soils, and suggest the prehistoric Rapa Nui population had extensive knowledge of how to overcome poor soil fertility, improve environmental conditions, and create a sustainable food supply. These activities demonstrate considerable adaptation and resilience to environmental challenges ‐ a finding that is inconsistent with an “ecocide” narrative.

## ACKNOWLEDGMENTS

We thank Jim Ehleringer, Thure Cerling, John M. Hayes and Alex Bentley for comments on an early draft of the paper, and to Janet Becker for assistance with error propagation calculations. We also thank Natasha Vokhshoori for her help analyzing the food samples and Oliver A. Chadwick, Peter M. Vitousek, and Katalyn Voss for providing soils samples from Anamarama. This research was partly funded by National Science Foundation Grant EF‐1137336 (to JR Ehleringer, TE Cerling, GJ Bowen, CC Miller and EM Riggs) through a Research in Residence Fellowship to CLJ at the University of Hawaii. Any opinions, findings, and conclusions or recommendations expressed in this material are those of the authors and do not necessarily reflect the views of the National Science Foundation. Additional funding for analytical costs was provided by a research grant to CLJ from the Kon‐Tiki Museum. CLJ would like to thank the Society for American Archaeology, whose 2015 Student Award donations included the ^14^C AMS dates from the Center for Applied Isotope Studies at the University of Georgia used in this study. The research was carried out while CLJ was supported by an Arts and Humanities Research Council PhD studentship (Award AH/K502947/1) at the University of Bristol. Funding for TL and analytical costs of food samples was provided by the DFG supported “The Future Ocean”. CLJ and BNP conceived and planned the project and measured the isotopic composition of human, faunal and totora reed samples. TL performed carbon isotope analyses of plant food samples and performed statistical analysis of data. TH, CL and RS provided archaeological samples and soils and contributed to data interpretation. NJW and CK‐L provided laboratory support and training in amino acid compound specific isotopic analyses, and NJW, CK‐L and HGC assisted CLJ with amino acid isotope sample analysis and contributed to data interpretation. CLJ and BNP wrote the manuscript and all authors revised and approved it. This is SOEST contribution number 10052.

## Supporting information

Supporting Information Appendix A.

Supporting Information Appendix B.
