# Supplementary material for: Diet of the prehistoric population of Rapa Nui (Easter Island, Chile) shows environmental adaptation and resilience
Source: Am J Phys Anthropol. 2017 Jun 30;164(2):343–61. doi: 10.1002/ajpa.23273 (PMC5637906; doi:10.1002/ajpa.23273)
Supplement: Supplementary file 1 — Supporting Information Appendix A. [file AJPA-164-343-s001.docx]

SUPPLEMETARY INFORMATION

Appendix A. Propagation of error calculations

Trophic position determined using equations 1 and 2 is uncertain. The measured values of δ^15^N*_glu_* and δ^15^N*_phe_* have inherent analytical uncertainty and there is uncertainty in compiled values of β.

$TP=\left(\frac{\delta^{15}N_{glu}-\delta^{15}N_{phe}+\beta}{\Delta_{glu-phe}}\right)+1$ eqn. S1

For any result w that is a function of three experimentally determined independent variables x, y and z, if the variance in x, y and z are known, they can be combined to calculate the variance in w following the general expression (e.g., Gelwicks and Hayes, 1990; Phillips and Gregg, 2001):

$\sigma_{w}^{2}=\left(\frac{\partial w}{\partial x}\right)^{2}\sigma_{x}^{2}+\left(\frac{\partial w}{\partial y}\right)^{2}\sigma_{y}^{2}+\left(\frac{\partial w}{\partial z}\right)^{2}\sigma_{z}^{2}$ eqn. S2

It follows that uncertainty in trophic position calculated using eqn. S1 can be determined by propagation of errors (e.g. Blum et al., 2013; Bradley et al., 2015) using the analytical solution of the differentiation of:

$\sigma_{TP}^{2}=\left(\frac{\partial TP}{\partial\delta^{15}N_{glu}}\right)^{2}\sigma_{\delta^{15}N_{glu}}^{2}+\left(\frac{\partial TP}{\partial\delta^{15}N_{phe}}\right)^{2}\sigma_{\delta^{15}N_{phe}}^{2}+\left(\frac{\partial TP}{\partial\beta}\right)^{2}\sigma_{\beta}^{2}+\left(\frac{\partial TP}{\partial\Delta_{glu-phe}}\right)^{2}\sigma_{\Delta_{glu-phe}}^{2}$ eqn. S3

The solution to equation S3 yields an expression for calculation of the standard deviation of the calculated trophic position for either a fully marine or fully terrestrial diet (Bradley et al. 2015).

$\sigma_{TP}=\sqrt{\left(\frac{1}{\Delta_{glu-phe}}\right)^{2}\sigma_{\delta^{15}N_{glu}}^{2}+\left(\frac{-1}{\Delta_{glu-phe}}\right)^{2}\sigma_{\delta^{15}N_{phe}}^{2}+\left(\frac{1}{\Delta_{glu-phe}}\right)^{2}\sigma_{\beta}^{2}+\left(\frac{-1}{\Delta_{glu-phe}^{2}}\left(\delta^{15}N_{glu}-\delta^{15}N_{phe}+\beta\right)\right)^{2}\sigma_{\Delta_{glu-phe}}^{2}}$ eqn. S4

Here we assumed that σ_β_*_marine_* is 0.9‰, σ_β_*_terrestrial_* is 1.6‰ and σ_Δ_ is 1.1‰ (Chikaraishi t al., 2009; 2010; 2011). The standard deviations for δ^15^N*_glu_* and δ^15^N*_phe_* values in our samples are based on replicate analyses at least in triplicate (Table S2).

Uncertainty in TP*_mixed_* can also be determined by propagation of errors using the analytical solution of partial differentiation of eqn. 3 and yields a measure of the standard deviation of calculated values of TP*_mixed_*:

$\sigma_{TP_{mixed}}=\sqrt{\left(\frac{1}{\Delta}\right)^{2}\sigma_{\delta^{15}N_{glu}}^{2}+\left(\frac{-1}{\Delta}\right)^{2}\sigma_{\delta^{15}N_{phe}}^{2}+\left(\frac{\beta_{m}-\beta_{t}}{\Delta}\right)^{2}\sigma_{f_{m}}^{2}+\left(\frac{1-f_{m}}{\Delta}\right)^{2}\sigma_{\beta_{t}}^{2}+\left(\frac{f_{m}}{\Delta}\right)^{2}\sigma_{\beta_{m}}^{2}+\left(\frac{-1}{\Delta^{2}}(\delta^{15}N_{glu}-\delta^{15}N_{phe}+(1-f_{m})\beta_{t}+f_{m}\beta_{m})\right)^{2}\sigma_{\Delta}^{2}}$ eqn. S5

In Equation S5, ƒ*_marine (phe)_* is abbreviated as ƒ*_m_* and β*_marine_* and β*_terrestrial_* are abbreviated as β*_m_* and β*_t_*, respectively. The variance of ƒ*_marine (phe)_* used in eqn. S5 is the propagated error calculated from Phillips and Gregg (2001).

Uncertainty in ƒ*_marine (HTL)_* was also determined by propagation of errors using partial differentiation of eqn. 4. The analytical solution of that partial differential equation yields a measure of the variance of calculated values of ƒ*_marine (HTL)_*:

$$\sigma_{f_{mmarine-(HTL)}}^{2}=\left(\frac{TP_{HTL}-1}{\beta_{m}-\beta_{t}}\right)^{2}\sigma_{\Delta_{glu-phe}}^{2}+\left(\frac{\Delta_{glu-phe}}{\beta_{m}-\beta_{t}}\right)^{2}\sigma_{TP_{HTL}}^{2}+\left(\frac{-1}{\beta_{m}-\beta_{t}}\right)^{2}\sigma_{\delta^{15}N_{glu}}^{2}+\left(\frac{1}{\beta_{m}-\beta_{t}}\right)^{2}\sigma_{\delta^{15}N_{phe}}^{2}$$

$+\left(\frac{1}{(\beta_{m}-\beta_{t})^{2}}(\Delta_{glu-phe}(TP_{HTL}-1)-\delta^{15}N_{glu}+\delta^{15}N_{phe}-\beta_{m})\right)^{2}\sigma_{\beta_{t}}^{2}+\left(\frac{-1}{(\beta_{m}-\beta_{t})^{2}}(\Delta_{glu-phe}(TP_{HTL}-1)-\delta^{15}N_{glu}+\delta^{15}N_{phe}-\beta_{t})\right)^{2}\sigma_{\beta_{m}}^{2}$ eqn. S6

In Equation S6, we abbreviate β*_marine_* and β*_terrestrial_* as β*_m_* and β*_t_*, respectively. We used the value of σ_HTL_ of 0.13 reported in Bonhommeau *et al.* (2013) in Equation S6.
