# Supplementary material for: Diet of the prehistoric population of Rapa Nui (Easter Island, Chile) shows environmental adaptation and resilience
Source: Am J Phys Anthropol. 2017 Jun 30;164(2):343–61. doi: 10.1002/ajpa.23273 (PMC5637906; doi:10.1002/ajpa.23273)
Supplement: Supplementary file 2 — Supporting Information Appendix B. [file AJPA-164-343-s002.docx]

Appendix B. Supplementary Tables

**Table S1:** List of all archaeological and modern reference material sampled for the current study, with location and stratigraphic data where available.

| Sample ID | **Site** | **Excavations** | **Context/Accession number** | **Type** | **Element(s)** |
| --- | --- | --- | --- | --- | --- |
| **RN001** | Ahu Nau Nau East | Kon-Tiki Museum 1986-1988 | 10N/0E 140-160cm | Terrestrial bird | Humerus |
| **RN002** | Ahu Nau Nau East | Kon-Tiki Museum 1986-1988 | 10N/0E 140-160cm | Bird | Long bone |
| **RN003** | Ahu Nau Nau East | Kon-Tiki Museum 1986-1988 | Const. 21, 7N/2W, 100-120cm | Rat | Femur |
| **RN004** | Ahu Nau Nau East | Kon-Tiki Museum 1986-1988 | Const. 21, 7N/2W, 100-120cm | Fish | Vertebra |
| **RN005** | Ahu Nau Nau East | Kon-Tiki Museum 1986-1988 | 9N/0E 120-140cm | Bird | Long bone |
| **RN006** | Ahu Nau Nau East | Kon-Tiki Museum 1986-1988 | 9N/0E 120-140cm | Marine mammal | Vertebra |
| **RN007** | Ahu Nau Nau East | Kon-Tiki Museum 1986-1988 | 4N/4W, 80-100cm | Fish | 2 x vertebrae |
| **RN008** | Ahu Nau Nau East | Kon-Tiki Museum 1986-1988 | 4N/4W, 80-100cm | Rat | Long bone |
| **RN009** | Ahu Nau Nau East | Kon-Tiki Museum 1986-1988 | 4N/4W, 80-100cm | Rat | Long bone |
| **RN010** | Ahu Nau Nau East | Kon-Tiki Museum 1986-1988 | 7N/2W 80-100cm | Bird | Long bone |
| **RN011** | Ahu Nau Nau East | Kon-Tiki Museum 1986-1988 | 7N/2W 80-100cm | Bird | Long bone |
| **RN012** | Ahu Nau Nau East | Kon-Tiki Museum 1986-1988 | 7N/2W 80-100cm | Marine mammal | Vertebral disc |
| **RN013** | Ahu Nau Nau East | Kon-Tiki Museum 1986-1988 | f17, N12/E0, 100-160cm | Bird | Long bone |
| **RN014** | Ahu Nau Nau East | Kon-Tiki Museum 1986-1988 | f17, N12/E0, 100-160cm | Rat | Femur |
| **RN015** | Ahu Nau Nau East | Kon-Tiki Museum 1986-1988 | f17, N12/E0, 100-160cm | Fish | Vertebra |
| **RN016** | Ahu Nau Nau East | Kon-Tiki Museum 1986-1988 | f20, 150-200cm | Bird | Furcula |
| **RN017** | Ahu Nau Nau East | Kon-Tiki Museum 1986-1988 | f20, 150-200cm | Rat | Long bone |
| **RN018** | Ahu Nau Nau East | Kon-Tiki Museum 1986-1988 | 4S/0E, 20-40 cm | Rat | Femur |
| **RN019** | Ahu Nau Nau East | Kon-Tiki Museum 1986-1988 | 11N/1E, 40-60cm | Rat | Femur |
| **RN020** | Ahu Nau Nau/Trench E | Kon-Tiki Museum 1986-1988 | Tr. E, sqm. 9-11, d 20-40cm | Whale | Fragments |
| **RN021** | Ahu Nau Nau/Trench E | Kon-Tiki Museum 1986-1988 | Tr. E, sqm. 14, d 80cm | Marine mammal | Loose fragments |
| **RN022** | Ahu Nau Nau/Trench E | Kon-Tiki Museum 1986-1988 | Tr. E, sqm. 3, d. 87cm | Rat | Humerus |
| **RN023** | Ahu Nau Nau/Trench E | Kon-Tiki Museum 1986-1988 | Tr. E, sqm. 10, d 40-60cm | Fish | Vertebra |
| **RN024** | Ahu Nau Nau/Trench E | Kon-Tiki Museum 1986-1988 | Tr. E, sqm. 10, d 40-60cm | Fish | Vertebra |
| **RN025** | Ahu Nau Nau/Trench C | Kon-Tiki Museum 1986-1988 | Tr. C, sqm. 8-11, d 290-300cm | Bird | Long bone |
| **RN026** | Ahu Tepeu E-13, grave 2 | Thor Heyerdahl 1956 | 1956-41-006 | Human | Phalanx |
| **RN028** | Ranu Raraku, statue 295 | Thor Heyerdahl 1956 | KTM 56-33-0091 | Human | Rib fragment |
| **RN032** | Ahu Nau Nau/Trench K | Kon-Tiki Museum 1986-1988 | KTM 88-2-0055 | Human | Rib fragment |
| **RN033** | Ahu Nau Nau/Trench K | Kon-Tiki Museum 1986-1988 | KTM 88-2-0017 | Human | Phalanx |
| **RN034** | Ahu Nau Nau/Trench K | Kon-Tiki Museum 1986-1988 | KTM 88-2-0042 | Human | Rib fragment |
| **RN035** | Ahu Nau Nau/Trench K | Kon-Tiki Museum 1986-1988 | KTM 88-4-0004 | Human | Rib fragment |
| **RN036** | Ahu Nau Nau/Trench K | Kon-Tiki Museum 1986-1988 | KTM 88-2-0217 | Human | Rib fragment |
| **RN037** | Ahu Nau Nau/Trench K | Kon-Tiki Museum 1986-1988 | KTM 88-2-0135 | Human | Rib fragment |
| **RN038** | Ahu Nau Nau/Trench K | Kon-Tiki Museum 1986-1988 | KTM 88-2-0132 | Human | Phalanx |
| **RN039** | Ahu Nau Nau/Trench K | Kon-Tiki Museum 1986-1988 | KTM 88-2-0098 | Human | Rib fragment |
| **RN040** | Ahu Nau Nau/Trench K | Kon-Tiki Museum 1986-1988 | KTM 88-2-0073 | Human | Rib fragment |
| **RN041** | Ahu Nau Nau/Trench K | Kon-Tiki Museum 1986-1988 | KTM 88-2-0082 | Human | Rib fragment |
| **RN047** | Ahu Tepeu E-13, grave 2 | Thor Heyerdahl 1956 | - | Totora reed | Fragment |
| **RN060** | Anakena | Terry Hunt and Carl Lipo | Collected from beneath prehistoric fallen road Moai | Archaeological Soil | - |
| **RN061** | Anakena | Terry Hunt and Carl Lipo | Collected from beneath prehistoric fallen road Moai | Archaeological Soil | - |
| **RN062** | Akahanga | Terry Hunt and Carl Lipo | Collected from beneath prehistoric fallen road Moai | Archaeological Soil | - |
| **RN063** | Vai Mata | Terry Hunt and Carl Lipo | Collected from beneath prehistoric fallen road Moai | Archaeological Soil | - |
| **RN064** | Vai Mata | Terry Hunt and Carl Lipo | Collected from beneath prehistoric fallen road Moai | Archaeological Soil | - |
| **RN070** | Ahu Nau Nau | Kon-Tiki Museum 1986-1988 | Trench A, Hor. 1.20m, Ver. 1.53m | Archaeological Soil | - |
| **RN071** | Vinapu | Kon-Tiki Museum 1986-1988 | Trench 7, Sample 2, Square 6 | Archaeological Soil | - |
| **RN072** | Ahu Nau Nau | Kon-Tiki Museum 1986-1988 | Trench C1, from floor, plaza level. Hor. 7.5m, Ver. 1.49-1.50 | Archaeological Soil | - |
| **RN073** | Anakena | Kon-Tiki Museum 1986-1988 | Trench M, Hor. 1-3m, Ver. 80-85cm | Palm nut endocarp | Fragments |
| **RN074** | Rapa Nui | Thor Heyerdahl 1956 | Unknown | Totora Reed | Fragments |
| **RN075** | Maitaki Te Moa | Terry Hunt and Carl Lipo | Outside manavai; ~1.5 m north of northern wall (Wofford 003B) | Modern Soil | - |
| **RN076** | Maitaki Te Moa | Terry Hunt and Carl Lipo | Inside manavai; ~0.2 m south of northern wall (Wofford 003D) | Modern Soil | - |
| **RN077** | Maitaki Te Moa | Terry Hunt and Carl Lipo | Inside manavai; approximately middle of manavai (Wofford 003E) | Modern Soil | - |
| **RN078** | Maitaki Te Moa | Terry Hunt and Carl Lipo | Inside manavai; ~0.2 m north of southern wall (Wofford 003F) | Modern Soil | - |
| **RN079** | Maitaki Te Moa | Terry Hunt and Carl Lipo | Outside manavai; ~2 m south of southern wall (Wofford 003H) | Modern Soil | - |
| **RN080** | Maitaki Te Moa | Terry Hunt and Carl Lipo | Outside manavai; ~3 m north of wall (Wofford 003I) | Modern Soil | - |
| **RN081** | Maitaki Te Moa | Terry Hunt and Carl Lipo | Outside manavai; 100 m west of manavai (Wofford 003J) | Modern Soil | - |
| **RN082** | Maitaki Te Moa | Terry Hunt and Carl Lipo | Outside manavai; ~2 m north of northern wall (Wofford 219B) | Modern Soil | - |
| **RN083** | Maitaki Te Moa | Terry Hunt and Carl Lipo | Inside manavai; ~0.1 south of northern wall (Wofford 219D) | Modern Soil | - |
| **RN084** | Maitaki Te Moa | Terry Hunt and Carl Lipo | Inside manavai; ~0.5 south of northern wall (Wofford 219E) | Modern Soil | - |
| **RN085** | Maitaki Te Moa | Terry Hunt and Carl Lipo | Inside manavai; ~0.5 m north of southern wall (Wofford 219F) | Modern Soil | - |
| **RN086** | Maitaki Te Moa | Terry Hunt and Carl Lipo | Outside manavai; ~2 m south of southern wall (Wofford 219H) | Modern Soil | - |
| **RN087** | Maitaki Te Moa | Terry Hunt and Carl Lipo | Outside manavai; 100 m west of manavai (Wofford 219J) | Modern Soil | - |
| **RN088** | Anakena | Terry Hunt and Carl Lipo | Outside manavai; ~1 m north of northern wall (Wofford 420A) | Modern Soil | - |
| **RN089** | Anakena | Terry Hunt and Carl Lipo | Outside manavai; ~0.1 m north of northern wall (Wofford 420C) | Modern Soil | - |
| **RN090** | Anakena | Terry Hunt and Carl Lipo | Inside manavai; ~0.3 m south of northern wall (Wofford 420D) | Modern Soil | - |
| **RN091** | Anakena | Terry Hunt and Carl Lipo | Inside manavai; approximately middle of manavai (Wofford 420E) | Modern Soil | - |
| **RN092** | Anakena | Terry Hunt and Carl Lipo | Inside manavai; ~0.2 m north of southern wall (Wofford 420F) | Modern Soil | - |
| **RN093** | Anakena | Terry Hunt and Carl Lipo | Outside manavai; ~0.2 m south of southern wall (Wofford 420G) | Modern Soil | - |
| **RN094** | Anakena | Terry Hunt and Carl Lipo | Outside manavai; ~3 m south of southern wall (Wofford 420I) | Modern Soil | - |
| **RN095** | Anakena | Terry Hunt and Carl Lipo | Outside manavai; 100 m west of manavai (Wofford 420J) | Modern Soil | - |
| **RN096** | Anakena | Terry Hunt and Carl Lipo | Outside manavai; ~2 m north of northernmost wall (Wofford 421A) | Modern Soil | - |
| **RN097** | Anakena | Terry Hunt and Carl Lipo | Outside manavai; ~0.2 m north of northernmost wall (Wofford 421C) | Modern Soil | - |
| **RN098** | Anakena | Terry Hunt and Carl Lipo | Inside north manavai; ~0.2 m south of northern wall (Wofford 421D) | Modern Soil | - |
| **RN099** | Anakena | Terry Hunt and Carl Lipo | Inside north manavai; ~1 m south of northern wall (Wofford 421E) | Modern Soil | - |
| **RN100** | Anakena | Terry Hunt and Carl Lipo | Inside south manavai; ~1 m south of middle wall (Wofford 421F) | Modern Soil | - |
| **RN101** | Anakena | Terry Hunt and Carl Lipo | Inside south manavai; ~0.2 m north of southern wall (Wofford 421G) | Modern Soil | - |
| **RN102** | Anakena | Terry Hunt and Carl Lipo | Outside manavai; ~3 m south of southernmost wall (Wofford 420I) | Modern Soil | - |
| **RN103** | Anakena | Terry Hunt and Carl Lipo | Outside manavai; 100 m west of manavai (Wofford 421K) | Modern Soil | - |
| **RN104** | Anamarama | Vitousek et al. (2014) | Inside rock garden; 34 m along transect (Vitousek et al. RN173) | Modern Soil |  |
| **RN105** | Anamarama | Vitousek et al. (2014) | Inside rock garden; 37 m along transect (Vitousek et al. RN175) | Modern Soil |  |
| **RN106** | Anamarama | Vitousek et al. (2014) | Inside rock garden; 40 m along transect (Vitousek et al. RN177) | Modern Soil |  |
| **RN107** | Anamarama | Vitousek et al. (2014) | Inside rock garden; 55 m along transect (Vitousek et al. RN187) | Modern Soil |  |
| **RN108** | Anamarama | Vitousek et al. (2014) | Outside rock garden; 76 m along transect (Vitousek et al. RN201) | Modern Soil |  |
| **RN109** | Anamarama | Vitousek et al. (2014) | Outside rock garden; 79 m along transect (Vitousek et al. RN203) | Modern Soil |  |
| **RN110** | Anamarama | Vitousek et al. (2014) | Outside rock garden; 82 m along transect (Vitousek et al. RN205) | Modern Soil |  |
| **RN111** | Anamarama | Vitousek et al. (2014) | Outside rock garden; 85 m along transect (Vitousek et al. RN207) | Modern Soil |  |
| **RN112** | Anamarama | Vitousek et al. (2014) | Inside rock garden; 125 m along transect (Vitousek et al. RN243) | Modern Soil |  |
| **RN113** | Anamarama | Vitousek et al. (2014) | Inside rock garden; 126 m along transect (Vitousek et al. RN245) | Modern Soil |  |
| **RN114** | Anamarama | Vitousek et al. (2014) | Inside rock garden; 127 m along transect (Vitousek et al. RN247) | Modern Soil |  |
| **RN115** | Anamarama | Vitousek et al. (2014) | Inside rock garden; 128 m along transect (Vitousek et al. RN249) | Modern Soil |  |
| **1149** | Anakena Dune | Terry Hunt 2004 | II - 2 | Rat | 2xtibia |
| **1287** | Anakena Dune | Terry Hunt 2004 | V - 6 | Rat | 1xfemur, 1xmandible, 1xtibia |
| **1292** | Anakena Dune | Terry Hunt 2004 | VI - 7 | Rat | 2xtibia |
| **1307** | Anakena Dune | Terry Hunt 2004 | VII - 8B | Rat | 1xinnominate |
| **1311** | Anakena Dune | Terry Hunt 2004 | VIII - 9 | Rat | 2xfemur |
| **1316** | Anakena Dune | Terry Hunt 2004 | IX - 10 | Rat | 1xtibia |
| **1276** | Anakena Dune | Terry Hunt 2004 | X - 23 | Rat | 1xfemur |
| **1328** | Anakena Dune | Terry Hunt 2004 | XII - 13 | Rat | 1xinnominate |
| **PF46** | Denmark | Modern | *Portula oleracea* | Common purslane | Leaf |
| **PF49** | Denmark | Modern | *Ipomoea batatas* | Sweet potato | Leaf |
| **PF63** | Sri Lanka | Modern | *Musa spp* | Banana, mini | Fruit |
| **PF74** | Nigeria | Modern | *Colocasia esculenta* | Taro | Tuber |
| **PF75** | Nigeria | Modern | *Dioscorea rotundata* | Yam | Tuber |
| **PF79** | Germany | Modern | *Brassica rapa* | Bok choy | Leaf |
| **PF80** | Ecuador | Modern | *Musa acuminata* | Red banana | Fruit |
| **PF84** | Spain | Modern | *Ipomoea batatas* | Sweet Potato | Tuber |
| **PF85** | Spain | Modern | *Ipomoea batatas* | Sweet Potato | Tuber |
| **PF94** | Puerto Rico | Modern | *Aiphanes minima* | Palm seed | Seed |
| **Ch** | Tropical North Pacific | Modern | *Coryphaena hippurus* | Mahimahi | Fish |
| **Xg** | Tropical North Pacific | Modern | *Xiphias gladius* | Swordfish | Fish |
| **Lg** | Tropical North Pacific | Modern | *Lampris guttatus* | Opah | Fish |
| **Mc1** | California 34.47 lat | Modern | *Mytilus californianus* | California mussel | Mussel |
| **Mc2** | California 33.20 lat | Modern | *Mytilus californianus* | California mussel | Mussel |
| **Mc3** | California 39.35 lat | Modern | *Mytilus californianus* | California mussel | Mussel |
| **Mc4** | California 42.00 lat | Modern | *Mytilus californianus* | California mussel | Mussel |
| **Mc5** | California 36.95 lat | Modern | *Mytilus californianus* | California mussel | Mussel |
| **HI Taro-1** | Oahu, Hawaii | Modern | *Colocasia esculenta* | Grown on fertilizer (δ^15^N = -0.6‰) | Tuber |
| **HI Taro-2** | Oahu, Hawaii | Modern | *Colocasia esculenta* | Grown on fertilizer (δ^15^N = -0.6‰) | Tuber |
| **HI Taro-3** | Oahu, Hawaii | Modern | *Colocasia esculenta* | Grown on fertilizer (δ^15^N = -0.6‰) | Tuber |
| **HI Taro-4** | Oahu, Hawaii | Modern | *Colocasia esculenta* | Grown on fertilizer (δ^15^N = -0.6‰) | Tuber |

**Table S2:** Bulk collagen, soil, and plant carbon and nitrogen concentrations and δ^13^C and δ^15^N values. For collagen, samples with C:N molar ratios above 3.6 were excluded from further study (see Methods).

| Sample ID | Type | μg N | δ^15^N  (‰ vs. AIR) | μg C | δ^13^C  (‰ vs. V-PDB) | C:N molar |
| --- | --- | --- | --- | --- | --- | --- |
| RN001 | Terrestrial bird | 44.2 | 9.8 | 125.5 | -20.0 | 3.3 |
| RN002 | Bird | 76.0 | 20.0 | 210.1 | -14.4 | 3.2 |
| RN003 | Rat | 66.9 | 8.2 | 189.0 | -18.2 | 3.3 |
| RN004 | Fish | 60.8 | 16.7 | 168.8 | -12.0 | 3.2 |
| RN005 | Bird | 72.8 | 14.3 | 203.2 | -13.0 | 3.3 |
| RN006 | Marine mammal | 54.9 | 14.7 | 151.0 | -11.8 | 3.2 |
| RN007 | Fish | 53.5 | 10.4 | 152.1 | -13.3 | 3.3 |
| RN008 | Rat | 70.9 | 13.2 | 200.4 | -17.2 | 3.3 |
| RN009 | Rat | 65.3 | 9.5 | 185.8 | -16.3 | 3.3 |
| RN010 | Bird | 73.1 | 12.5 | 204.6 | -13.2 | 3.3 |
| RN011 | Bird | 68.8 | 14.3 | 191.3 | -12.0 | 3.2 |
| RN012 | Marine mammal | 47.8 | 15.9 | 131.4 | -12.3 | 3.2 |
| RN014 | Rat | 72.8 | 12.1 | 207.1 | -19.8 | 3.3 |
| RN016 | Bird | 70.5 | 17.1 | 195.3 | -13.4 | 3.2 |
| RN017 | Rat | 51.4 | 13.6 | 149.8 | -19.0 | 3.4 |
| RN018 | Rat | 54.9 | 13.0 | 157.0 | -9.6 | 3.3 |
| RN019 | Rat | 76.8 | 13.8 | 217.2 | -18.1 | 3.3 |
| RN020 | Whale | 69.1 | 8.0 | 194.6 | -17.9 | 3.3 |
| RN021 | Marine mammal | 64.3 | 15.1 | 179.1 | -12.0 | 3.3 |
| RN022 | Rat | 50.5 | 15.0 | 142.3 | -18.6 | 3.3 |
| RN023 | Fish | 67.3 | 8.1 | 186.2 | -12.2 | 3.2 |
| RN024 | Fish | 52.1 | 10.8 | 152.7 | -15.1 | 3.4 |
| RN025 | Bird | 71.3 | 15.1 | 198.3 | -13.2 | 3.2 |
| RN026 | Human | 79.2 | 12.4 | 216.5 | -18.3 | 3.2 |
| RN028 | Human | 32.3 | 14.0 | 105.2 | -18.9 | 3.8 |
| RN032 | Marine mammal | 77.0 | 15.5 | 212.9 | -11.5 | 3.2 |
| RN033 | Human | 75.8 | 14.8 | 208.8 | -17.9 | 3.2 |
| RN034 | Human | 59.0 | 14.9 | 162.2 | -18.9 | 3.2 |
| RN035 | Human | 61.9 | 15.3 | 169.7 | -19.1 | 3.2 |
| RN036 | Human | 74.8 | 13.5 | 201.3 | -19.7 | 3.1 |
| RN037 | Human | 76.9 | 15.4 | 215.8 | -18.9 | 3.3 |
| RN038 | Human | 78.8 | 15.5 | 218.3 | -18.5 | 3.2 |
| RN039 | Human | 81.8 | 14.0 | 228.5 | -18.1 | 3.3 |
| RN040 | Human | 59.4 | 14.9 | 166.9 | -17.8 | 3.3 |
| RN041 | Human | 61.4 | 14.9 | 172.3 | -18.5 | 3.3 |
| RN047 | Totora reed | 76.6 | 22.6 | 500.9 | -26.6 | 7.6 |
| RN060 | Soil | 124.7 | -0.2 | 1581.3 | -19.2 | 14.8 |
| RN061 | Soil | 125.7 | -0.3 | 1611.6 | -19.1 | 15.0 |
| RN062 | Soil | 130.0 | 0.6 | 1688.1 | -19.1 | 15.2 |
| RN063 | Soil | 115.9 | 1.3 | 1479.8 | -19.0 | 15.0 |
| RN064 | Soil | 121.2 | 1.0 | 1561.5 | -19.1 | 15.0 |
| RN070 | Soil | 17.1 | 11.3 | 145.2 | -22.7 | 9.9 |
| RN071 | Soil | 12.6 | 11.2 | 180.2 | -18.3 | 16.7 |
| RN072 | Soil | 16.8 | 9.7 | 150.7 | -23.6 | 10.5 |
| RN073 | Palm nut endocarp | 15.3 | 8.8 | 1073.1 | -21.9 | 81.8 |
| RN074 | Totora reed | 21.7 | 2.3 | 1923.8 | -26.1 | 103.4 |
| RN075 | Soil | 32.1 | 11.8 | 373.1 | -14.4 | 13.6 |
| RN076 | Soil | 29.0 | 10.0 | 341.7 | -24.7 | 13.7 |
| RN077 | Soil | 27.5 | 10.8 | 307.3 | -23.7 | 13.0 |
| RN078 | Soil | 34.9 | 10.7 | 406.5 | -20.8 | 13.6 |
| RN079 | Soil | 29.6 | 8.5 | 331.4 | -14.5 | 13.1 |
| RN080 | Soil | 32.7 | 10.7 | 330.6 | -14.4 | 11.8 |
| RN081 | Soil | 27.9 | 10.5 | 285.1 | -14.3 | 11.9 |
| RN082 | Soil | 26.0 | 10.9 | 271.8 | -17.4 | 12.2 |
| RN083 | Soil | 29.2 | 12.0 | 286.7 | -20.5 | 11.5 |
| RN084 | Soil | 31.9 | 9.9 | 330.2 | -19.5 | 12.1 |
| RN085 | Soil | 30.9 | 11.2 | 289.5 | -19.9 | 10.9 |
| RN086 | Soil | 28.2 | 10.1 | 292.6 | -17.3 | 12.1 |
| RN087 | Soil | 30.9 | 9.0 | 342.7 | -16.9 | 12.9 |
| RN088 | Soil | 24.8 | 9.3 | 310.5 | -17.2 | 14.6 |
| RN089 | Soil | 31.9 | 9.4 | 378.7 | -16.8 | 13.9 |
| RN090 | Soil | 27.1 | 8.6 | 321.5 | -17.8 | 13.8 |
| RN091 | Soil | 34.5 | 9.8 | 415.1 | -17.2 | 14.0 |
| RN092 | Soil | 31.8 | 9.5 | 390.6 | -18.5 | 14.3 |
| RN093 | Soil | 28.1 | 8.3 | 347.0 | -17.5 | 14.4 |
| RN094 | Soil | 31.1 | 8.2 | 385.2 | -16.7 | 14.5 |
| RN095 | Soil | 27.2 | 8.0 | 323.0 | -17.7 | 13.9 |
| RN096 | Soil | 34.1 | 6.8 | 412.0 | -15.1 | 14.1 |
| RN097 | Soil | 25.3 | 7.6 | 300.0 | -14.7 | 13.8 |
| RN098 | Soil | 32.8 | 10.1 | 342.3 | -20.8 | 12.2 |
| RN099 | Soil | 21.6 | 10.1 | 226.7 | -18.6 | 12.2 |
| RN100 | Soil | 19.5 | 10.2 | 179.3 | -23.2 | 10.7 |
| RN101 | Soil | 28.5 | 10.1 | 304.1 | -22.3 | 12.4 |
| RN102 | Soil | 23.4 | 7.6 | 257.2 | -16.8 | 12.8 |
| RN103 | Soil | 27.2 | 9.2 | 258.5 | -16.5 | 11.1 |
| RN104 | Soil | 41.4 | 10.5 | 413.2 | -21.3 | 11.6 |
| RN105 | Soil | 32.8 | 10.6 | 303.4 | -21.1 | 10.8 |
| RN106 | Soil | 35.2 | 10.2 | 322.1 | -20.0 | 10.7 |
| RN107 | Soil | 34.6 | 9.8 | 370.9 | -19.1 | 12.5 |
| RN108 | Soil | 20.2 | 11.8 | 166.4 | -18.7 | 9.6 |
| RN109 | Soil | 24.1 | 10.6 | 217.6 | -17.5 | 10.5 |
| RN110 | Soil | 20.1 | 10.4 | 190.4 | -17.2 | 11.1 |
| RN111 | Soil | 21.6 | 10.8 | 196.6 | -18.3 | 10.6 |
| RN112 | Soil | 29.9 | 10.5 | 284.7 | -20.8 | 11.1 |
| RN113 | Soil | 35.2 | 10.0 | 367.9 | -18.7 | 12.2 |
| RN114 | Soil | 40.7 | 9.1 | 464.4 | -17.3 | 13.3 |
| RN115 | Soil | 39.9 | 9.3 | 452.7 | -17.1 | 13.2 |
| 1149 | Rat | 70.6 | 13.0 | 197.5 | -13.3 | 3.3 |
| 1287 | Rat | 76.0 | 14.3 | 203.8 | -18.7 | 3.1 |
| 1292 | Rat | 74.7 | 14.7 | 208.6 | -13.7 | 3.3 |
| 1307 | Rat | 83.8 | 8.2 | 230.6 | -17.0 | 3.2 |
| 1311 | Rat | 75.5 | 13.6 | 218.8 | -18.9 | 3.4 |
| 1316 | Rat | 57.5 | 13.6 | 157.7 | -19.2 | 3.2 |
| 1276 | Rat | 67.7 | 15.3 | 189.2 | -15.3 | 3.3 |
| 1328 | Rat | 70.1 | 9.8 | 196.2 | -17.0 | 3.3 |
| HI Taro-1 | Taro | 75.8 | 4.4 | 831.3 | -26.9 | 12.8 |
| HI Taro-2 | Taro | 72.7 | 5.0 | 847.4 | -26.5 | 13.6 |
| HI Taro-3 | Taro | 85.0 | 1.8 | 795.7 | -26.7 | 10.9 |
| HI Taro-4 | Taro | 69.6 | 3.1 | 808.6 | -26.2 | 15.2 |

**Table S3:** δ^15^N amino acid data. Values presented are the average of triplicate runs, with standard deviations in brackets. Abbreviations: Ala = alanine, Gly = Glycine, Thre = Threonine, Ser = Serine, Val = Valine, Leu = Leucine, Ile = Isoleucine, Pro = Proline, Phe = Phenylalanine, Tyr = Tyrosine, Lys = Lysine, Asx = Aspartic Acid and Aspartamine, Glx = Glutamic Acid and Glutamine. (The terminal amide groups in glutamine (Gln) and aspartamine (Asn) are cleaved during the chemical isolation of amino acids, and as a result these amino acids are converted to glutamic acid (Glu) and aspartic acid (Asp), respectively. Thus, the isotope value of a combined Glu + Gln (termed Glx) and a combined Asn + Asp (termed Asx) are measured.)

| ID | Type | Ala | Gly | Thr | Ser | Val | Leu | Ile | Pro | Asx | Glx | Phe | Tyr | Lys |
| --- | --- | --- | --- | --- | --- | --- | --- | --- | --- | --- | --- | --- | --- | --- |
| 1307 | Rat | 8.77 (0.63) | 3.93 (0.68) | -5.81 (0.37) | 9.25 (0.58) | 9.94 (0.24) | 9.99 (0.3) | - | 11.49 (0.27) | 9.13 (0.27) | 12.17 (0.45) | 7.19 (0.13) | 15.6 (0.99) | 2.69 (0.44) |
| 1328 | Rat | 14.45 (0.37) | 9.86 (0.09) | 0.19 (0.78) | 15.03 (0.45) | 16.25 (0.11) | 16.02 (0.34) | - | 17.89 (0.09) | 15.02 (0.05) | 17.54 (0.32) | 12.99 (0.59) | 21.77 (0.22) | 9.84 (0.42) |
| RN001 | Bird (terr.) | 11.8 (0.21) | 9.77 (0.14) | -5.1 (0.17) | 10.19 (0.37) | 12.35 (0.06) | 12.17 (0.25) | 11.82 (0.04) | 12.58 (0.04) | 12.97 (0.37) | 12.94 (0.13) | 10.12 (0.14) | 20.19 (0.93) | 6.45 (0.32) |
| RN002 | Bird | 23.69 (0.38) | 16.46 (0.07) | -31.99 (0.91) | 15.71 (0.4) | 27.42 (0.83) | 26.2 (0.35) | - | 27.14 (0.26) | 21.54 (0.53) | 24.96 (0.4) | 6.4 (0.3) | 28.24 (0.21) | 9.73 (0.13) |
| RN007 | Fish | 21.48 (0.41) | -0.13 (0.18) | -21.78 (0.47) | 5.41 (0.12) | 15.81 (0.78) | 18.46 (0.85) | - | 17.05 (0.33) | 16.52 (0.46) | 21.0 (0.36) | 0.41 (0.62) | 15.87 (0.9) | 6.1 (0.19) |
| RN010 | Bird | 16.9 (0.21) | 7.42 (0.23) | -26.79 (0.25) | 8.6 (0.19) | 14.71 (0.87) | 17.23 (0.34) | - | 17.64 (0.17) | 14.49 (0.4) | 18.67 (0.05) | 1.45 (0.41) | 20.22 (0.69) | 4.17 (0.16) |
| RN014 | Rat | 11.03 (0.35) | 5.45 (0.72) | -4.75 (0.49) | 9.95 (0.77) | 12.41 (0.39) | 11.28 (0.34) | - | 15.27 (0.24) | 12.11 (0.51) | 15.0 (0.22) | 10.89 (0.54) | 17.75 (0.31) | 4.99 (0.15) |
| RN020 | Whale | 13.2 (0.48) | 0.54 (0.09) | -23.31 (1.03) | 4.63 (0.5) | 12.62 (0.67) | 13.28 (0.49) | 13.66 (0.61) | 13.71 (0.01) | 11.23 (0.41) | 14.24 (0.56) | 0.71 (0.48) | 12.27 (0.69) | 3.69 (0.54) |
| RN023 | Fish | 12.07 (0.6) | 2.72 (0.54) | -2.09 (0.45) | 4.56 (0.41) | 10.05 (0.3) | 11.28 (0.36) |  | 8.77 (0.68) | 11.39 (0.72) | 13.11 (0.3) | 1.65 (0.53) | 13.19 (0.86) | 4.15 (0.47) |
| RN026 | Human | 12.14 (0.43) | 9.36 (0.01) | -5.49 (0.31) | 12.41 (0.06) | 13.89 (0.71) | 14.57 (0.38) | 13.16 (0.21) | 17.98 (0.11) | 13.18 (0.2) | 15.27 (0.54) | 10.52 (0.34) | 21.93 (0.52) | 5.95 (0.63) |
| RN032 | Marine mammal | 26.23 (0.14) | 5.25 (0.08) | -30.58 (0.56) | 12.35 (0.27) | 20.18 (0.94) | 26.83 (0.53) | 25.33 (0.29) | 23.6 (0.32) | 22.6 (0.05) | 28.69 (0.36) | 8.73 (0.31) | - | 7.56 (0.34) |
| RN033 | Human | 13.26 (0.27) | 11.66 (0.25) | -5.71 (0.28) | 15.66 (0.12) | 16.87 (0.38) | 16.11 (0.37) | - | 17.96 (0.08) | 14.16 (0.25) | 17.11 (0.3) | 11.83 (0.79) | 19.12 (0.22) | 8.5 (0.42) |
| RN035 | Human | 15.15 (0.26) | 12.69 (0.27) | -3.99 (0.76) | 14.22 (0.3) | 18.68 (0.79) | 16.86 (0.35) | 17.5 (0.85) | 19.54 (0.12) | 15.79 (0.15) | 20.63 (0.27) | 14.01 (0.36) | 25.28 (0.48) | 10.52 (0.87) |
| RN036 | Human | 13.21 (0.12) | 11.67 (0.99) | -6.26 (0.4) | 12.12 (0.14) | 15.54 (0.92) | 14.64 (0.32) | - | 17.82 (0.08) | 13.2 (0.76) | 15.95 (0.11) | 10.12 (0.54) | 21.17 (0.58) | 6.45 (0.23) |
| RN037 | Human | 16.38 (0.45) | 11.82 (0.39) | -8.19 (0.67) | 14.38 (0.52) | 16.19 (0.96) | 18.49 (0.67) | - | 20.03 (0.14) | 15.34 (0.66) | 18.93 (0.08) | 11.69 (0.18) | 20.85 (0.39) | 6.01 (0.44) |
| RN038 | Human | 16.03 (0.32) | 13.93 (0.14) | -5.32 (0.97) | 17.61 (0.68) | 18.57 (0.29) | 17.94 (0.27) | - | 20.82 (0.09) | 17.34 (0.72) | 19.74 (0.5) | 12.21 (0.77) | 22.1 (0.94) | 8.03 (0.77) |
| RN039 | Human | 14.09 (0.98) | 10.73 (0.33) | -12.97 (1.33) | 12.52 (0.47) | 16.97 (0.17) | 15.98 (0.16) | 16.48 (0.37) | 18.65 (0.16) | 14.6 (0.39) | 20.4 (0.32) | 11.88 (0.09) | 24.92 (1.54) | 8.26 (0.75) |
| RN040 | Human | 15.01 (0.41) | 11.44 (0.25) | -10.77 (0.28) | 14.93 (0.31) | 17.07 (0.79) | 16.24 (0.07) | - | 19.95 (0.12) | 15.7 (0.22) | 17.76 (0.24) | 10.82 (0.3) | 22.08 (0.81) | 8.9 (0.06) |
| RN041 | Human | 15.53 (0.67) | 12.38 (0.45) | -9.14 (0.49) | 13.45 (0.13) | 15.87 (0.68) | 15.58 (0.38) | - | 20.45 (0.04) | 15.06 (0.49) | 17.13 (0.1) | 10.85 (0.28) | 22.33 (0.23) | 6.33 (0.38) |
| RN047 | Totora reed | 26.49 (0.33) | 21.45 (0.13) | 16.68 (0.16) | 21.23 (0.09) | 29.03 (1.46) | 19.50 (0.47) | 21.08 (0.50) | 20.76 (0.15) | 24.09 (0.26) | 23.81 (0.21) | 22.70 (0.91) | - | 16.62 (0.47) |

**Table S4:** δ^13^C amino acid data. Values presented are the average of triplicate runs, with standard deviations in brackets. Abbreviations: Ala = alanine, Gly = Glycine, Thre = Threonine, Ser = Serine, Val = Valine, Leu = Leucine, Ile = Isoleucine, Pro = Proline, Phe = Phenylalanine, Tyr = Tyrosine, Lys = Lysine, Asx = Aspartic Acid and Aspartamine, Glx = Glutamic Acid and Glutamine. (The terminal amide groups in glutamine (Gln) and aspartamine (Asn) are cleaved during the chemical isolation of amino acids, and as a result these amino acids are converted to glutamic acid (Glu) and aspartic acid (Asp), respectively. Thus, the isotope value of a combined Glu + Gln (termed Glx) and a combined Asn + Asp (termed Asx) are measured.) *Samples were measured with the NACME method in the Larsen lab.

| **ID** | **Type** | Ala | Asx | Glx | Gly | Ile | Leu | Lys | Met | Phe | Pro | Ser | Thr | Tyr | Val |
| --- | --- | --- | --- | --- | --- | --- | --- | --- | --- | --- | --- | --- | --- | --- | --- |
| **1149** | Rat | -15.79 (1.02) | -13.86 (1.07) | -15.09 (0.41) | -10.03 (0.87) | -18.28 (1.50) | -24.46 (0.15) | -19.60 (0.57) | -20.10 (0.98) | -19.15 (0.29) | -14.22 (0.21) | -9.45 (0.26) | -16.17 (0.77) | -10.02 (0.35) | -24.49 (0.51) |
| **1276** | Rat | -15.74 (0.28) | -15.20 (0.37) | -16.28 (0.32) | -9.81 (0.49) | -22.48 (1.55) | -28.14 (0.50) | -22.83 (0.51) | -24.75 (0.30) | -24.11 (0.52) | -16.44 (0.37) | -10.80 (0.88) | -21.36 (0.70) | -14.70 (0.35) | -26.60 (0.36) |
| **1287** | Rat | -21.53 (0.51) | -18.25 (0.91) | -19.76 (4.20) | -13.76 (1.49) | -21.49 (0.29) | -30.16 (0.28) | -22.24 (0.87) | - | -24.82 (1.12) | -18.17 (0.45) | -15.38 (0.78) | -23.11 (1.01) | -12.01 (1.06) | -27.01 (0.36) |
| **1292** | Rat | -17.73 (1.14) | -15.03 (0.80) | -13.36 (0.50) | -10.88 (0.77) | -18.32 (0.92) | -25.05 (0.22) | -17.78 (0.25) | -20.15 (1.17) | -20.49 (0.39) | -15.09 (0.22) | -9.48 (1.04) | -19.32 (1.06) | -9.23 (0.66) | -25.20 (0.08) |
| **1307** | Rat | -20.58 (0.26) | -18.41 (0.30) | -17.29 (1.68) | -12.06 (0.64) | -22.26 (0.78) | -29.19 (0.18) | -22.55 (1.35) | -22.85 (0.93) | -24.33 (0.48) | -17.44 (0.22) | -10.90 (1.37) | -24.20 (0.92) | -12.61 (0.63) | -27.40 (0.26) |
| **1311** | Rat | -20.20 (0.44) | -18.07 (0.64) | -18.44 (2.27) | -13.67 (0.86) | -23.36 (0.41) | -29.92 (0.21) | -21.30 (0.45) | -27.11 (1.24) | -24.21 (0.29) | -18.77 (0.26) | -15.22 (0.83) | -19.33 (1.11) | -13.98 (0.66) | -27.59 (0.64) |
| **1316** | Rat | -23.87 (1.23) | -19.16 (0.83) | -18.68 (0.23) | -17.48 (0.36) | -23.01 (0.68) | -30.91 (0.54) | -22.93 (0.78) | -24.61 (0.56) | -25.48 (0.60) | -20.11 (0.36) | -17.72 (0.47) | -20.42 (1.18) | -12.60 (0.87) | -28.11 (0.61) |
| **1328** | Rat | -21.98 (0.94) | -19.23 (1.02) | -18.36 (0.84) | -14.70 (4.02) | -24.07 (0.67) | -30.51 (0.33) | -22.72 (0.29) | -27.61 (1.32) | -24.41 (0.30) | -19.36 (0.72) | -17.86 (0.45) | -21.32 (0.88) | -14.54 (0.33) | -28.86 (0.45) |
| **RN002** | Bird | -23.32 (0.21) | -18.68 (0.14) | -18.00 (0.21) | -11.24 (0.25) | - | -25.85 (0.23) | -18.66 (0.19) | -16.72 (0.40) | -22.94 (0.11) | -15.76 (0.19) | -8.25 (0.13) | -13.49 (0.58) | -13.76 (0.33) | -23.30 (0.40) |
| **RN007** | Fish | -16.08 (0.63) | -16.33 (0.34) | -14.24 (0.24) | -4.01 (0.75) | - | -23.24 (0.39) | -17.32 (0.34) | -19.42 (0.78) | -21.45 (0.25) | -14.53 (0.42) | -6.54 (0.58) | -13.36 (0.27) | -10.65 (0.33) | -22.69 (0.53) |
| **RN020** | Whale | -23.60 (0.23) | -23.78 (0.17) | -23.95 (0.42) | -11.75 (0.03) | -19.72 (0.26) | -30.87 (0.13) | -22.12 (0.64) | - | -26.55 (0.41) | -20.55 (0.18) | -6.24 (0.45) | -13.90 (0.46) | -12.35 (0.46) | -27.03 (0.29) |
| **RN023** | Fish | -15.06 (0.37) | -13.10 (0.20) | -11.35 (0.25) | -7.09 (0.92) | -15.07 (0.20) | -20.37 (0.02) | -13.61 (0.04) | -16.42 (0.40) | -20.18 (0.19) | -14.37 (0.18) | 0.96 (0.24) | -10.63 (0.66) | -7.50 (0.13) | -19.15 (0.54) |
| **RN026** | Human | -19.37 (0.25) | -22.56 (0.31) | -19.99 (0.31) | -17.33 (0.36) | -20.13 (0.10) | -28.25 (0.24) | -21.03 (0.26) | - | -24.67 (0.32) | -18.06 (0.30) | -8.67 (0.88) | -12.00 (0.82) | -12.34 (0.33) | -25.87 (0.63) |
| **RN032** | M. mammal | -11.28 (0.52) | -12.20 (0.72) | -11.18 (0.24) | -1.42 (0.12) | -14.85 (0.52) | -21.34 (0.54) | -14.52 (0.38) | - | -21.15 (0.27) | -11.99 (0.27) | 2.13 (0.40) | -8.52 (0.58) | -7.00 (0.59) | -17.91 (0.66) |
| **RN033** | Human | -22.18 (0.07) | -22.67 (0.44) | -21.67 (0.51) | -17.13 (0.74) | - | -28.58 (0.18) | -20.47 (0.37) | - | -23.37 (0.35) | -19.06 (0.50) | -12.31 (0.54) | -12.94 (0.76) | -16.13 (0.34) | -26.15 (0.51) |
| **RN035** | Human | -18.32 (0.30) | -17.83 (0.14) | -18.51 (0.29) | -13.73 (0.34) | -22.32 (0.61) | -29.38 (0.07) | -20.75 (0.24) | - | -24.32 (0.22) | -18.18 (0.20) | -10.85 (0.07) | -13.72 (0.28) | -12.25 (0.22) | -26.32 (0.21) |
| **RN036** | Human | -20.84 (0.12) | -22.80 (1.09) | -20.43 (1.15) | -19.01 (0.78) | - | -30.24 (0.33) | -21.46 (0.06) | - | -24.82 (0.31) | -18.97 (0.50) | -13.73 (0.79) | -17.21 (0.65) | -16.84 (0.23) | -28.58 (0.56) |
| **RN037** | Human | -19.78 (0.33) | -19.90 (0.09) | -17.83 (0.26) | -16.34 (0.26) | -20.72 (0.49) | -27.45 (0.02) | -18.66 (0.11) | - | -23.39 (0.07) | -17.08 (0.08) | -11.93 (0.42) | -12.59 (0.06) | -13.46 (0.43) | -24.67 (0.12) |
| **RN039** | Human | -18.42 (0.30) | -19.25 (0.23) | -17.12 (0.06) | -13.97 (0.12) | -20.51 (0.35) | -27.40 (0.26) | -19.39 (0.16) | - | -23.96 (0.22) | -17.26 (0.26) | -10.69 (0.50) | -12.39 (0.16) | -13.51 (0.45) | -24.34 (0.19) |
| **RN041** | Human | -18.61 (0.23) | -20.29 (0.20) | -17.70 (0.21) | -15.08 (0.86) | -20.68 (0.08) | -27.73 (0.25) | -19.23 (0.36) | - | -23.71 (0.22) | -17.19 (0.30) | -11.36 (0.11) | -12.85 (0.82) | -14.09 (0.30) | -25.80 (0.40) |
| **PF46*** | Common purslane | -13.28 (0.11) | -7.98 (0.31) | -13.50 (0.03) | -13.19 (0.21) | -16.73 (0.38) | -23.94 (0.37) | -12.09 (0.39) | -16.46 (0.46) | -17.98 (0.08) | -14.86 (0.27) | -4.70 (0.35) | -3.89 (0.37) | -17.75 (0.02) | -22.71 (0.17) |
| **PF49*** | Sweet potato | -26.11 (0.05) | -21.78 (0.16) | -28.02 (0.10) | -24.59 (0.11) | -29.52 (0.24) | -36.49 (0.05) | -23.54 (0.08) | -31.43 (0.24) | -28.93 (0.02) | -28.14 (0.13) | -26.69 (0.27) | -14.68 (0.12) | -29.78 (0.01) | -33.79 (0.07) |
| **PF63*** | Banana, mini | -24.28 (0.15) | -24.60 (0.23) | -28.84 (0.06) | - | -28.40 (0.31) | -34.89 (0.20) | -25.51 (0.43) | -29.47 (0.13) | -29.37 (0.25) | -28.37 (0.13) | -17.00 (0.35) | -11.62 (0.25) | -28.61 (0.15) | -32.57 (0.18) |
| **PF74*** | Taro | -26.37 (0.04) | -25.77 (0.14) | -28.21 (0.04) | -23.15 (0.28) | -30.18 (0.28) | -34.12 (0.17) | -25.31 (0.18) | -33.12 (0.19) | -29.86 (0.21) | -28.02 (0.22) | -19.34 (0.22) | -16.57 (0.23) | -31.00 (0.23) | -32.86 (0.05) |
| **PF75*** | Yam | -30.22 (0.09) | -26.25 (0.29) | -29.64 (0.04) | - | -31.88 (0.14) | -37.71 (0.20) | -25.89 (0.19) | -33.14 (0.17) | -32.03 (0.09) | -28.36 (0.21) | -17.91 (0.59) | -16.85 (0.09) | -31.62 (0.09) | -34.41 (0.07) |
| **PF79*** | Bok choy | -31.23 (0.40) | -27.07 (0.42) | -30.51 (0.03) | - | -32.09 (0.08) | -38.19 (0.15) | -30.26 (0.35) | - | -33.08 (0.13) | -31.83 (0.41) | -16.93 (0.42) | -19.10 (0.11) | -34.77 (0.37) | -37.14 (0.19) |
| **PF80*** | Red banana | -24.90 (0.46) | -19.91 (0.73) | -28.71 (0.08) | -19.51 (0.34) | -29.52 (0.26) | -36.06 (0.47) | -25.76 (0.25) | -31.85 (0.49) | -29.42 (0.02) | -26.29 (NA) | -15.69 (NA) | -13.06 (NA) | -29.10 (0.14) | -32.80 (0.07) |
| **PF84*** | Sweet Potato | -27.61 (0.15) | -23.18 (0.46) | -27.43 (0.17) | - | -27.13 (0.58) | -34.82 (0.18) | -24.18 (0.39) | -32.64 (0.35) | -30.23 (0.15) | -27.14 (0.21) | -16.47 (0.25) | -17.08 (0.12) | -30.44 (0.09) | -32.99 (0.16) |
| **PF85*** | Sweet Potato | -26.11 (0.18) | -23.07 (0.84) | -27.81 (0.23) | -23.48 (0.68) | -29.61 (0.25) | -35.58 (0.47) | -23.98 (0.54) | -31.53 (0.22) | -30.73 (0.29) | -27.29 (0.30) | -15.27 (0.19) | -18.62 (0.74) | -31.13 (0.43) | -33.11 (0.24) |
| **PF94*** | Aiphanes minima | -28.5 (0.6) | -20.4 (0.6) | -26.7 (0.2) |  | -27.2 (0.7) | -30.4 (0.2) | -21.0 (0.4) | -23.8 (0.5) | -27.1 (0.2) | -25.8 (0.7) | -13.7 (1.2) | -14.5 (1.0) | -28.0 (0.1) | -30.0 (0.1) |
| **Mc*** | Mahimahi | -17.60 (0.13) | -19.54 (0.30) | -16.27 (0.02) | -12.60 (0.12) | -20.53 (0.38) | -25.51 (0.14) | -18.36 (0.04) | -25.41 (0.07) | -27.91 (0.15) | -18.21 (0.12) | -8.96 (0.18) | -13.22 (0.16) | -26.62 (0.09) | -23.48 (0.13) |
| **Xg*** | Swordfish | -20.72 (0.12) | -15.26 (0.03) | -19.11 (0.07) | -12.70 (0.23) | -19.97 (1.10) | -26.57 (0.28) | -19.55 (0.16) | -28.34 (0.21) | -28.82 (0.18) | -18.93 (0.12) | -10.92 (0.19) | -13.24 (0.30) | -27.47 (0.01) | -24.40 (0.09) |
| **Lg*** | Opah | -20.80 (0.03) | -18.20 (0.48) | -19.45 (0.11) | -10.85 (0.19) | -20.98 (0.21) | -27.12 (0.26) | -19.64 (0.18) | -25.78 (0.06) | -28.25 (0.20) | -18.51 (0.16) | -10.44 (0.20) | -10.71 (0.09) | -27.69 (0.18) | -23.87 (0.08) |
| **Mc1*** | California mussel | -13.05 (0.10) | -15.95 (2.10) | -13.18 (0.00) | -11.95 (0.10) | -18.58 (0.10) | -25.08 (0.10) | -14.28 (0.10) | -17.33 (0.30) | -24.15 (0.10) | - | - | -12.91 (0.20) | -22.75 (0.10) | -23.18 (0.10) |
| **Mc2*** | California mussel | -13.54 (0.10) | -15.98 (0.20) | -13.64 (0.00) | -10.99 (0.10) | -18.92 (0.00) | -26.11 (0.10) | -15.15 (0.00) | -17.67 (0.10) | -24.68 (0.00) | - | - | -12.30 (0.10) | -23.19 (0.10) | -24.41 (0.10) |
| **Mc3*** | California mussel | -15.27 (0.10) | -15.90 (0.20) | -15.16 (0.10) | -7.24 (0.10) | -19.24 (0.00) | -26.09 (0.10) | -15.55 (0.20) | -18.03 (0.10) | -24.57 (0.00) | - | - | -11.41 (0.20) | -23.81 (0.00) | -23.53 (0.00) |
| **Mc4*** | California mussel | -13.55 (0.00) | -14.71 (0.10) | -13.65 (0.10) | -8.88 (0.10) | -17.37 (0.10) | -25.02 (0.10) | -13.36 (0.10) | -16.04 (0.10) | -23.38 (0.10) | - | - | -10.52 (0.40) | -22.39 (0.10) | -22.40 (0.20) |
| **Mc5*** | California mussel | -12.27 (0.00) | -14.21 (0.10) | -12.01 (0.10) | -6.83 (0.00) | -16.79 (0.00) | -23.40 (0.00) | -13.05 (0.00) | -15.79 (0.10) | -22.47 (0.10) | - | - | -10.99 (0.30) | -21.26 (0.10) | -21.51 (0.10) |

**Table S5:** Relative contributions of marine and terrestrial protein sources to consumers. The relative contributions were calculated with the Bayesian based software FRUITS using mean normalized δ^13^C values of leucine, lysine, phenylalanine and valine (Table S4). Marine-I comprised of samples RN002, RN007, RN023, RN032, Ch, Lg, and Xg, Marine-II comprised of Mc1, Mc2, Mc3, Mc4, and Mc5; Plant-I of PF46, PF49 PF75, PF84, PF85 and PF94; Plant-II of PF63, PF74, PF79, and PF80. See Table S1 for sample identities.

| **ID** | **Type** | **Food** | **Mean** | **sd** | **2.5pc** | **median** | **97.5pc** |
| --- | --- | --- | --- | --- | --- | --- | --- |
| 1292 | Rats | Marine-I | 0.4031 | 0.2063 | 0.02548 | 0.4381 | 0.7525 |
| 1292 | Rats | Marine-II | 0.01652 | 0.01539 | 5.03E-04 | 0.01211 | 0.05814 |
| 1292 | Rats | Plant-I | 0.1604 | 0.1218 | 0.006031 | 0.1327 | 0.4422 |
| 1292 | Rats | Plant-II | 0.4201 | 0.2656 | 0.0159 | 0.4037 | 0.898 |
| 1307 | Rats | Marine-I | 0.3777 | 0.1342 | 0.09077 | 0.3843 | 0.6259 |
| 1307 | Rats | Marine-II | 0.1075 | 0.07359 | 0.005359 | 0.09543 | 0.2745 |
| 1307 | Rats | Plant-I | 0.1759 | 0.1209 | 0.00763 | 0.1575 | 0.4404 |
| 1307 | Rats | Plant-II | 0.3389 | 0.168 | 0.02552 | 0.3465 | 0.6395 |
| 1276 | Rats | Marine-I | 0.3802 | 0.1608 | 0.07179 | 0.3822 | 0.687 |
| 1276 | Rats | Marine-II | 0.1658 | 0.1012 | 0.009814 | 0.1582 | 0.3786 |
| 1276 | Rats | Plant-I | 0.1865 | 0.1291 | 0.007284 | 0.1663 | 0.4692 |
| 1276 | Rats | Plant-II | 0.2675 | 0.1549 | 0.01527 | 0.2591 | 0.5762 |
| 1149 | Rats | Marine-I | 0.1175 | 0.08318 | 0.006634 | 0.103 | 0.3149 |
| 1149 | Rats | Marine-II | 0.0666 | 0.05225 | 0.002341 | 0.05508 | 0.1936 |
| 1149 | Rats | Plant-I | 0.05698 | 0.0488 | 0.00185 | 0.04381 | 0.1811 |
| 1149 | Rats | Plant-II | 0.759 | 0.09047 | 0.5671 | 0.7653 | 0.9169 |
| 1328 | Rats | Marine-I | 0.1747 | 0.09484 | 0.01602 | 0.1712 | 0.3778 |
| 1328 | Rats | Marine-II | 0.1056 | 0.07218 | 0.005031 | 0.094 | 0.2663 |
| 1328 | Rats | Plant-I | 0.329 | 0.1921 | 0.02016 | 0.3179 | 0.6953 |
| 1328 | Rats | Plant-II | 0.3907 | 0.2002 | 0.0316 | 0.394 | 0.7532 |
| 1311 | Rats | Marine-I | 0.225 | 0.1078 | 0.02496 | 0.2243 | 0.4434 |
| 1311 | Rats | Marine-II | 0.1664 | 0.1062 | 0.008444 | 0.1539 | 0.3939 |
| 1311 | Rats | Plant-I | 0.3562 | 0.1709 | 0.0323 | 0.3726 | 0.6394 |
| 1311 | Rats | Plant-II | 0.2524 | 0.1681 | 0.01084 | 0.2299 | 0.5963 |
| 1316 | Rats | Marine-I | 0.2354 | 0.1405 | 0.0149 | 0.2233 | 0.533 |
| 1316 | Rats | Marine-II | 0.211 | 0.1203 | 0.01485 | 0.2047 | 0.4556 |
| 1316 | Rats | Plant-I | 0.2889 | 0.1655 | 0.01771 | 0.2842 | 0.6042 |
| 1316 | Rats | Plant-II | 0.2647 | 0.1679 | 0.01215 | 0.2498 | 0.6059 |
| 1287 | Rats | Marine-I | 0.2156 | 0.1429 | 0.01234 | 0.1948 | 0.5349 |
| 1287 | Rats | Marine-II | 0.252 | 0.1081 | 0.03102 | 0.2609 | 0.4458 |
| 1287 | Rats | Plant-I | 0.27 | 0.1545 | 0.01513 | 0.2692 | 0.5533 |
| 1287 | Rats | Plant-II | 0.2624 | 0.166 | 0.01167 | 0.2489 | 0.5915 |
| RN033 | Humans | Marine-I | 0.2714 | 0.1186 | 0.04418 | 0.274 | 0.5019 |
| RN033 | Humans | Marine-II | 0.1842 | 0.1126 | 0.009837 | 0.1748 | 0.4221 |
| RN033 | Humans | Plant-I | 0.2994 | 0.1557 | 0.01858 | 0.309 | 0.5669 |
| RN033 | Humans | Plant-II | 0.2449 | 0.1613 | 0.01189 | 0.2263 | 0.5772 |
| RN036 | Humans | Marine-I | 0.2782 | 0.1074 | 0.06276 | 0.2808 | 0.4889 |
| RN036 | Humans | Marine-II | 0.1104 | 0.0852 | 0.003976 | 0.09208 | 0.3142 |
| RN036 | Humans | Plant-I | 0.3528 | 0.1747 | 0.02969 | 0.3669 | 0.6477 |
| RN036 | Humans | Plant-II | 0.2586 | 0.1733 | 0.01132 | 0.2356 | 0.6178 |
| RN035 | Humans | Marine-I | 0.3098 | 0.1024 | 0.1253 | 0.3063 | 0.5169 |
| RN035 | Humans | Marine-II | 0.1822 | 0.08946 | 0.01876 | 0.1839 | 0.3511 |
| RN035 | Humans | Plant-I | 0.3031 | 0.1472 | 0.02265 | 0.3235 | 0.5307 |
| RN035 | Humans | Plant-II | 0.205 | 0.1425 | 0.00718 | 0.1837 | 0.4953 |
| RN037 | Humans | Marine-I | 0.3404 | 0.05374 | 0.2389 | 0.3384 | 0.435 |
| RN037 | Humans | Marine-II | 0.1595 | 0.07434 | 0.01321 | 0.1699 | 0.2911 |
| RN037 | Humans | Plant-I | 0.2261 | 0.168 | 0.002063 | 0.2052 | 0.4983 |
| RN037 | Humans | Plant-II | 0.2741 | 0.1747 | 0.006827 | 0.2971 | 0.553 |
| RN039 | Humans | Marine-I | 0.5199 | 0.1159 | 0.3124 | 0.5129 | 0.7563 |
| RN039 | Humans | Marine-II | 0.1873 | 0.09947 | 0.01412 | 0.1853 | 0.3785 |
| RN039 | Humans | Plant-I | 0.1615 | 0.09061 | 0.01101 | 0.1599 | 0.3319 |
| RN039 | Humans | Plant-II | 0.1313 | 0.09186 | 0.004956 | 0.118 | 0.3293 |
| RN041 | Humans | Marine-I | 0.4813 | 0.09288 | 0.2984 | 0.4811 | 0.6673 |
| RN041 | Humans | Marine-II | 0.1017 | 0.07479 | 0.00412 | 0.08764 | 0.2795 |
| RN041 | Humans | Plant-I | 0.2443 | 0.1241 | 0.01859 | 0.2516 | 0.4673 |
| RN041 | Humans | Plant-II | 0.1727 | 0.1197 | 0.006454 | 0.1541 | 0.4321 |
| RN026 | Humans | Marine-I | 0.4645 | 0.1394 | 0.1808 | 0.4689 | 0.7231 |
| RN026 | Humans | Marine-II | 0.2122 | 0.1368 | 0.009809 | 0.1968 | 0.5142 |
| RN026 | Humans | Plant-I | 0.1635 | 0.1015 | 0.008563 | 0.1549 | 0.3711 |
| RN026 | Humans | Plant-II | 0.1598 | 0.104 | 0.007841 | 0.1471 | 0.3828 |
